# Developing an optimal stratification model for colorectal cancer screening and reducing racial disparities in multi-center population-based studies

**DOI:** 10.1186/s13073-024-01355-y

**Published:** 2024-06-13

**Authors:** Jianbo Tian, Ming Zhang, Fuwei Zhang, Kai Gao, Zequn Lu, Yimin Cai, Can Chen, Caibo Ning, Yanmin Li, Sangni Qian, Hao Bai, Yizhuo Liu, Heng Zhang, Shuoni Chen, Xiangpan Li, Yongchang Wei, Bin Li, Ying Zhu, Jinhua Yang, Mingjuan Jin, Xiaoping Miao, Kun Chen

**Affiliations:** 1 Department of Epidemiology and Biostatistics, School of Public Health, Department of Gastrointestinal Oncology, Zhongnan Hospital of Wuhan University, TaiKang Center for Life and Medical Sciences, Wuhan University, Wuhan, 430071 China; 2 Research Center of Public Health, Renmin Hospital of Wuhan University, Wuhan University, Wuhan, 430071 China; 3 Department of Epidemiology and Biostatistics, School of Public Health, Zhejiang University School of Medicine, Hangzhou, China; 4 Department of Colorectal Surgery and Oncology, The Second Affiliated Hospital, Zhejiang University School of Medicine, Hangzhou, China; 5 Department of Epidemiology and Biostatistics, School of Public Health, Wuhan University, Wuhan, China; 6 Department of Gastrointestinal Oncology, Hubei Cancer Clinical Study Center, Zhongnan Hospital of Wuhan University, Wuhan, China; 7 Jiashan Institute of Cancer Prevention and Treatment, Jiashan, China; 8 Jiangsu Collaborative Innovation Center for Cancer Personalized Medicine, Nanjing Medical University, Nanjing, China

**Keywords:** CRC early screening, Colorectal neoplasm, Polygenic risk score, Lifestyle factors, Trans-ancestry

## Abstract

**Background:**

Early detection of colorectal neoplasms can reduce the colorectal cancer (CRC) burden by timely intervention for high-risk individuals. However, effective risk prediction models are lacking for personalized CRC early screening in East Asian (EAS) population. We aimed to develop, validate, and optimize a comprehensive risk prediction model across all stages of the dynamic adenoma-carcinoma sequence in EAS population.

**Methods:**

To develop precision risk-stratification and intervention strategies, we developed three trans-ancestry PRSs targeting colorectal neoplasms: (1) using 148 previously identified CRC risk loci (PRS_148_); (2) SNPs selection from large-scale meta-analysis data by clumping and thresholding (PRS_183_); (3) PRS-CSx, a Bayesian approach for genome-wide risk prediction (PRS_Genomewide_). Then, the performance of each PRS was assessed and validated in two independent cross-sectional screening sets, including 4600 patients with advanced colorectal neoplasm, 4495 patients with non-advanced adenoma, and 21,199 normal individuals from the ZJCRC (Zhejiang colorectal cancer set; EAS) and PLCO (the Prostate, Lung, Colorectal, and Ovarian Cancer Screening Trial; European, EUR) studies. The optimal PRS was further incorporated with lifestyle factors to stratify individual risk and ultimately tested in the PLCO and UK Biobank prospective cohorts, totaling 350,013 participants.

**Results:**

Three trans-ancestry PRSs achieved moderately improved predictive performance in EAS compared to EUR populations. Remarkably, the PRSs effectively facilitated a thorough risk assessment across all stages of the dynamic adenoma-carcinoma sequence. Among these models, PRS_183_ demonstrated the optimal discriminatory ability in both EAS and EUR validation datasets, particularly for individuals at risk of colorectal neoplasms. Using two large-scale and independent prospective cohorts, we further confirmed a significant dose–response effect of PRS_183_ on incident colorectal neoplasms. Incorporating PRS_183_ with lifestyle factors into a comprehensive strategy improves risk stratification and discriminatory accuracy compared to using PRS or lifestyle factors separately. This comprehensive risk-stratified model shows potential in addressing missed diagnoses in screening tests (best NPV = 0.93), while moderately reducing unnecessary screening (best PPV = 0.32).

**Conclusions:**

Our comprehensive risk-stratified model in population-based CRC screening trials represents a promising advancement in personalized risk assessment, facilitating tailored CRC screening in the EAS population. This approach enhances the transferability of PRSs across ancestries and thereby helps address health disparity.

**Graphical Abstract:**

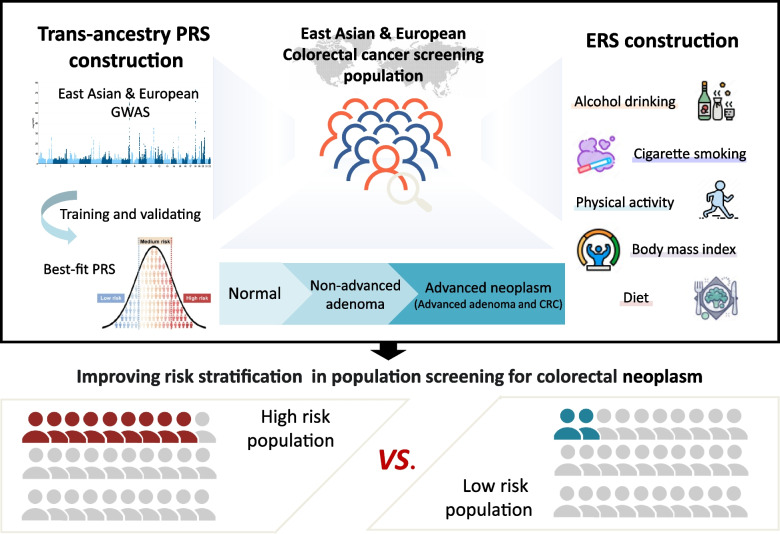

**Supplementary Information:**

The online version contains supplementary material available at 10.1186/s13073-024-01355-y.

## Background

Colorectal cancer (CRC) is the third most common cancer worldwide and the second most common cause of cancer-related death [1]. While the incidence of CRC is stabilizing or even declining in highly developed countries, it is increasing rapidly in developing countries, particularly in East Asian (EAS) [2]. This disparity is largely explained by different levels of colorectal cancer screening implementation [3]. Colorectal cancer develops through precursor stages over 10 to 15 years, allowing a considerable screening window to detect early cancer and precursor lesions [4]. Among the screening tools, colonoscopy is regarded as the gold standard for detecting CRC and precancerous neoplasms and proven effective in reducing CRC incidence and mortality [5]. However, most developing countries in EAS still lack effective form of CRC screening programs, because screening in a huge population is cost-prohibitive and requires the capabilities of local doctors and access to available technology. Furthermore, there remains a substantial under- and over-utilization of CRC screening with associated harms. Thus, risk-based screening represents as the first step towards a more feasible and cost-effective screening strategy by redistributing screening resources from low-risk to high-risk individuals.

Genome-wide association studies (GWASs) have identified more than 200 loci associated with CRC risk [6–9]. Polygenic risk score (PRS) based on GWAS aggregates genetic effects across the genome to measure the overall genetic liability to a trait or disease. Thus far, most PRSs for CRC have been established specifically in European ancestry, showing promise in identifying individuals at high risk of developing CRC [10]. Based on the largest and most recent European (EUR) ancestry CRC GWAS, PRS using 140 genetic variants achieved a discovery population AUC of 0.629 [11]. However, a notable disparity persists in the utilization of colorectal cancer PRSs within the EAS population, mainly due to limited research and accuracy in predictions [12]. A recent study constructed an EAS-EUR PRS to effectively stratify CRC risk [12], yet this PRS primarily centered on clinically diagnosed CRC cases, inadvertently potentially missing the chance to detect early-stage neoplasm [13]. Notably, including different stages along the dynamic adenoma-carcinoma sequence from the screening population into the PRS will facilitate the development of a more comprehensive colorectal neoplasm risk assessment model for the detection of early-stage lesions.

Many studies have shown that PRSs based on European training data attain lower accuracy when applied to populations of non-European ancestry [14]. Additionally, PRSs based on non-European training data may overcome these limitations but are currently suffered from much smaller training sample sizes. To address this issue, Ruan et al. [15] proposed a trans-ancestry PRS model, PRS-CSx, via a shared continuous shrinkage prior to couple SNP effects across populations. They attained average 75% relative improvement in prediction accuracy for schizophrenia in Asian cohorts compared to prediction models from a single-ancestry GWAS. Moreover, additional methods, such as prioritizing causal variants using functional genomic annotations or integrating GWAS summary statistics from multiple populations in a multivariate model, have been demonstrated to improve trans-ancestry genetic prediction [16]. Therefore, while striving to expand East Asian sample representation in CRC GWAS, exploring various trans-ancestry PRS methods can further enhance the accuracy of CRC prediction models within the EAS population.

Adherence to healthy lifestyle has been demonstrated to reduce CRC risk, particularly when tailored to individuals at high risk [17]. Recent studies have shown that combination of lifestyle and genetic factors may substantially improve prediction of CRC risk [18]. However, there was few studies to integrated both aspects to predicting the early-stage colorectal neoplasm in a true screening setting. It is essential to incorporate both PRS and lifestyle factors into the risk predictive model to further enhance the effectiveness of personalized CRC screening strategies.

To bridge these gaps, we utilized multiple independent large-scale GWASs data and provided a framework for optimizing a trans-ancestry PRS model encompassing EAS and EUR populations. By incorporating PRS with lifestyle factors, we established a comprehensive risk-stratified approach targeting individuals across different stage of the dynamic adenoma-carcinoma sequence. This comprehensive risk-stratified strategy contributes to stratifying populations based on colorectal neoplasms risk, serving as a guide for personalized screening to promote health equity across different ethnicities.

## Methods

### Characteristics of the study population in the validation and testing stage

To develop a trans-ancestry PRS model for colorectal neoplasm, we used the Zhejiang colorectal cancer (ZJCRC) case–control set as the assessment set, ZJCRC cross-sectional screening set, and the Prostate, Lung, Colorectal, and Ovarian Cancer Screening Trial (PLCO) cross-sectional screening set as the East Asian and European validation set respectively. We further tested in PLCO incident adenoma cohort and UK Biobank cohort (Additional file 1: Table S1-6).

Five thousand nine hundred eighty subjects from ZJCRC case–control set and ZJCRC cross-sectional screening set were genotyped using the Illumina™ Asian Screening Array (ASA) system (Additional file 2: Figure S1, Additional file 1: Table S7). Following principal component analysis, eight outlier samples were excluded, leaving a final analysis cohort of 5972 Chinese individuals.

#### ZJCRC case–control set

Participants were recruited from a one-to-one matched case–control study conducted from 2015 to 2022 based on an ongoing population-based CRC screening program running in Jiashan County, Zhejiang Province, China. The recruitment and diagnostic criteria and inclusion and exclusion criteria were previously reported [19, 20]. Demographic and lifestyle information was collected from questionnaires survey or direct measurement including age, sex, family history of CRC, and lifestyle habits. Written informed consent was obtained from all the study subjects. The disease stage classification was in accordance with the World Health Organization (WHO) classification standard. Each participant had undergone a high-quality colonoscopy examination. A total of 1814 patients with advanced colorectal neoplasm (including 1622 advanced adenoma and 192 cancer cases) and 1814 lesion-free controls were finally included in this study. All controls were matched to the cases by gender and age (5 years) in a 1:1 ratio. There was no significant racial difference between the sample groups in these sample sets (Additional file 2: Figure S2).

#### ZJCRC cross-sectional screening set

Participants were recruited from a cross-sectional screening study performed in 2014, as a part of the early work on the CRC screening program conducted in Jiashan County. More information about the data collection process is available in our previous report [21]. One hundred twenty-three patients with advanced colorectal neoplasm (including 108 advanced adenoma and 15 cancer cases), 549 patients with non-advanced adenoma, and 1672 normal individuals without any finding by screening colonoscopy were included. There was no significant racial difference between the sample groups in these sample sets (Additional file 2: Figure S2).

### Genotyping and imputation

Subjects from ZJCRC case–control set and ZJCRC cross-sectional screening set were genotyped using the Illumina™ Asian Screening Array (ASA) system to identify potential susceptibility variants. The case and control samples were mixed and randomly allocated in the plates. All initial genotyping reactions of cases and controls were performed simultaneously on the same platform, and researchers performing the assays were blinded to the case/control status. Genotype calling and quality control procedures were performed according to a standard protocol.

Preparation of genotype data included pre-imputation quality control (QC), imputation, and post-imputation QC. All genetic data were performed with stringent quality control to exclude low-quality samples and SNPs. Genotype data of the ZJCRC case–control set and ZJCRC cross-sectional screening set were imputed through the Michigan Imputation Server with the 1000 Genomes Project Phase III data as a reference. To obtain high-quality genotypes, strict criteria were applied to filter out low-quality variants: (1) SNPs on sex chromosomes (33,342 variants); (2) SNPs with call-rate < 95% (17 variants); (3) SNPs with minor allele frequency < 0.1% (905,917 variants); (4) SNPs that failed the Hardy–Weinberg equilibrium test with a *P*-value < 10^−6^ (91,360 variants). After imputation, we obtained 9,361,599 genotyped or imputed autosomal SNPs. Eight outlier samples were filtered out by principal component analysis.

The imputation methods and more details of the PLCO and UK Biobank populations are available on the PLCO (https://cdas.cancer.gov/plco/) and UK Biobank (biobank.ctsu.ox.ac.uk/crystal/refer.cgi?id = 157,020) official website. Genotype and quality control procedures for each study can be found in the supplement (Additional file 1: Table S7).

#### PLCO cross-sectional screening set

The Prostate, Lung, Colorectal, and Ovarian (PLCO) Cancer Screening Trial is a randomized, controlled trial aiming to assess the effectiveness of screening for prostate, lung, colorectal, and ovarian cancer [22]. Detailed information is presented on its official website. Our analysis included participants who underwent flexible sigmoidoscopy in the intervention arm of the trial collected up to 2022. Cases were those who had a negative baseline trial screen and were discovered to have adenoma or cancer in the colon or rectum at T3/T5. Controls were those with negative trial screens for adenoma at both baseline and T3/T5. Participants without genetic data were excluded from the study. Finally, 2663 patients with advanced colorectal neoplasm, 3946 patients with non-advanced adenoma, and 17,713 normal individuals without finding at screening colonoscopy were included in our cross-sectional retrospective study.

### Public cohorts of testing

#### PLCO incident adenoma cohort

According to the inclusion criteria in the PLCO study, the PLCO incident adenoma cohort included participants who had a negative screen at baseline and had either a negative screen at T3/T5 or a positive screen at T3/T5 with a left-sided adenoma found on follow-up to the screen, as part of PLCO Cancer Screening Trial. Detailed information on the cohort can be found on the official website. Participants who had a diagnosis of CRC or colorectal polyps at baseline and participants with missing genotype data or covariates at baseline were excluded. Finally, 369 patients with advanced colorectal neoplasm, 701 patients with non-advanced adenoma were found, and 14,922 normal individuals without finding at colonoscopy during follow-up.

#### UK Biobank cohort

UK Biobank is a large-scale database and cohort containing genetic and health information with follow-up from more than 500,000 UK participants [23]. All participants included were free of cancer at baseline. CRC cases were defined as subjects with newly primary invasive CRC diagnosed from CRC according to ICD10 (C180, C182-C189, C19, C20) codes, and patients with ICD-O-3 tumor histology codes 8240–8249, 9590–9729 were excluded. Since the colonoscopy information is not collected, advanced adenoma cases were defined as primary in situ CRC cases according to ICD10 (D010-D012) codes or benign neoplasms according to ICD10 codes (D120, D122, D123, D124-D128, D374, D375) with ICD-O-3 tumor histology codes 8210, 8211, 8220, 8221, or 8261–8263 [11]. Participants with missing genotype data or covariates at baseline or a history of other cancers according to the first cancer diagnosis or history of therapy on other cancers were excluded. Finally, 2980 patients with advanced colorectal neoplasm (including 2749 CRC cases and 231 advanced adenoma cases) were found, and there were 331,041 cancer-free normal individuals during follow-up.

### Construction methods of trans-ancestry PRS

#### Approach 1: PRS based on known EUR and EAS SNPs associated with CRC (PRS_148_)

We collected 148 independent SNPs significantly associated with CRC (*P* < 5.0 × 10^−8^) from large CRC GWASs conducted in East Asians (53 SNPs), the European-ancestry population (89 SNPs), and both populations (6 SNPs) by searching the literature (Additional file 1: Table S9) [24, 25]. All studies that were published on or before February 25, 2021 were also included. We calculate the sum of the weights of SNPs to construct the PRS based on the formula $PRS={\sum }_{i=1}^{n}{\beta }_{i}{SNP}_{i}$, in which *n* means the number of SNPs (0, 1, or 2), means the number of the risk alleles for the *i-*th SNP, and means the effect size of the risk alleles.

#### Approach 2: PRS with SNP selection by clumping and thresholding (PRS_183_)

We performed a meta-analysis of GWAS data for Asian and European populations. Based on the summary statistics from the meta-analysis of EAS and EUR GWAS, we built a PRS applying a clumping and thresholding method. Specifically, for any pair of SNPs with a distance smaller than 250 kb and a series of different LD values, the less significant SNP is removed in our study. Subsequently, we generated polygenic risk scores with different significance *P*-value thresholds via the software PLINK-1.9 (Additional file 1: Table S8, S10).

#### Approach 3: PRS derived from whole genome by PRS-CSx (PRS_Genomewide_)

We derived genome-wide PRS via PRS-CSx on the basis of GWAS summary data from UK Biobank and BioBank Japan as EUR and EAS GWAS statistics. PRS-CSx is a Bayesian multigene model building and prediction framework via a shared continuous shrinkage prior that was developed to enhance cross-population PRS prediction by integrating GWAS generalized statistics from multiple ethnicities [15]. Here, we restricted the analysis to HapMap3 SNPs as suggested. The final PRS-CSx output included 998,609 variants for polygenic risk score calculation.

### *Meta*-analysis of GWAS data from European and Asian populations

In approach 2, inverse variance-weighted fixed-effects meta-analysis was performed with METAL based on the GWAS summary statistics from EAS (BioBank Japan-CRC GWAS; 7062 cases and 195,745 controls) and EUR populations (UK Biobank, FinnGen, and CORECT CRC GWASs; total including 15,714 cases and 621,182 controls) [26]. The beta value of each individual study was matched to a common allele for each SNP. Genomic control correction was applied. SNPs that had significant heterogeneity (*P* value for heterogeneity test < 0.001) and were not present in EAS and EUR populations were excluded. Finally, a subset of 6,993,745 SNPs was retained for subsequent analysis. For details of quality control information, see Additional file 2: Figure S1.

## The public GWAS summary statistics for the *meta*-analysis

### European GWAS

#### UK Biobank-CRC GWAS

Several GWAS have been conducted based on the available population genotype data of the UK biobank. The GWAS summary statistics we used were sourced from the MRC Integrative Epidemiology Unit (IEU) Open GWAS database (https://gwas.mrcieu.ac.uk/). Five thousand six hundred fifty-seven cases and 377,673 cancer-free controls were included after strictly filtering. For more details, please refer to the official website.

#### FinnGen-CRC GWAS

The FinnGen study contains over 500,000 genome information with digital health care data from Finland [27]. The GWAS summary statistics for all traits included in the study are accessible and downloadable via the public website (https://www.finngen.fi/en/access_results). Four thousand nine hundred fifty-seven cases and 238,678 cancer-free controls were included.

#### CORECT-CRC GWAS

The Colorectal Cancer Transdisciplinary (CORECT) is composed of 6 observational studies of colorectal cancer: (1) Molecular Epidemiology of Colorectal Cancer Study, (2) Colon Cancer Family Registry Study, (3) Kentucky Case–Control Study, (4) American Cancer Society CPS II nested case–control study, (5) Melbourne nested case–control study, and (6) Newfoundland case–control study. Genotype data were obtained from dbGaP (http://www.ncbi.nlm.nih.gov/dbgap, accession numbers phs001856 and phs001499) [28]. A total of 5100 cases and 4831 controls were retained for analysis.

### East Asian GWAS

#### BioBank Japan-CRC GWAS

BioBank Japan (BBJ) has recruited a total of 260,000 patients and 51 diseases in Japan, of which large-scale GWAS was performed and made public on the website (http://jenger.riken.jp/). Seven thousand sixty-two cases and 195,745 controls were included in the CRC GWAS study.

#### Construction of environmental risk score (ERS)

Five modifiable lifestyle risk factors associated with CRC were included in the ERS construction: cigarette smoking, alcohol drinking, less physical activity, unhealthy diet, and high body weight. Further details on the specific criteria for each indicator and questionnaire data collection of the CRC screening program have been described previously [19]. Cigarette smoking was divided into current, former, and never smoking. Current smoking was defined as consuming at least one cigarette per day for more than 1 year or consuming over 300 cigarettes within 3 months, and former smoking was defined as quitting smoking for more than 6 months prior to colonoscopy. Alcohol drinking was also divided into current, former, and never drinking. Current drinking was defined as consuming ≥ 100 g of any alcohol per week, and former drinking was defined as quitting drinking for more than 6 months prior to colonoscopy. Physical activity was referred to any aerobic exercise for > 30 min (such as running, cycling, brisk walking, etc.) and further divided into ≤ 4 times and > 4 times per week based on the frequency. A short qualitative food frequency questionnaire was used to assess the frequency of dietary intake per week over the past year. Dietary quality score with a maximum of eight points was constructed to reflect the adherence to a healthy diet. Red meat or processed meat intake was rated negatively, whereas fresh fruit or vegetable intake was rated positively. Body mass index (BMI) was derived from measured weight and height and categorized according to the cut-offs from the guideline for the prevention and control of overweight and obesity in Chinese adults. For each of the five lifestyle factors, we further defined a binary criterion, by which the participants received a score of 1 if they met the following criterion or 0 otherwise: never smoking, never drinking, physical activity > 4 times/week, adhering to a healthy diet (dietary quality score ≥ 5), having a healthy weight (BMI < 24 kg/m^2^). Missing data were imputed by sex and age-specific predictive mean matching. Each factor was divided into 0 for healthy behavior and 1 for unhealthy behavior. In order to create a new ERS for screening, the multivariate logistic regression model was employed in the assessment set. Other potential confounding factors such as age, sex, and family history were adjusted in the model. And the estimated weight of each lifestyle factor represents a proportional increase in the risk of colorectal neoplasms. The estimated weight for each modifiable environmental factor was then summed to form an ERS for each participant (Additional file 1: Table S11). Due to the high missing rate of physical activity data, only four lifestyle factors except physical activity were included in PLCO cross-sectional screening set and PLCO incident adenoma cohort. Conditional logistic regression was used to examine the association between different stages of neoplasm in the assessment and validation set. The summary of participants of the different environmental factors in each population is shown in Additional file 1: Table S12-16.

### ATAC-seq

ATAC assay was performed on CRC tissues by SeqHealth (Wuhan, China). In brief, 500 mg tissue was treated with cell lysis buffer, and nucleus was collected by centrifuging for 10 min at 500 g at 4 °C. Transposition and high-throughput DNA sequencing library was carried out by TruePrep DNA Library Prep Kit V2 for Illumina kit (Vazyme, China). The library products were enriched, quantified, and finally sequenced on Novaseq 6000 sequencer (Illumina) with PE150 model. Raw sequencing data was first filtered by Trimmomatic (v.0.36), low-quality reads were discarded, and the reads contaminated with adaptor sequences were trimmed. Clean Reads were further treated with FastUniq (v.1.1) to eliminate duplication. Deduplicated reads were then mapped to the human reference genome using bowtie2 (v.2.2.6) with default parameters. Afterwards, we processed the data to generate BAM files with samtools (v.1.12) and made intersect between 10 CRC biosamples with bedtools (v.2.27.1).

### Hi-C and Hi-C data processing

The Hi-C libraries include crosslinking, chromatin digestion with four-cutter restriction enzyme MboI and marking of DNA ends, ligation and purification, shearing, and biotin pull down. A Hi-C map is a matrix of DNA-DNA contacts produced by the Hi-C experiment. The valid pairs after pooling were binned into 200 kb (100, 40, 20, 10, 5 kb) nonoverlapping genomic intervals to generate contact maps. Raw Hi-C contact maps can contain many different biases, such as map-ability, GC content, and uneven distribution of restriction enzyme sites. The corresponding cumulative probability *P*-values and FDR *q*-values were calculated in the Ay’s Fit-Hi-C software for contacts between 5 kb bins for intrachromosomal interactions, and the interactions with *q*-values less than 0.1 were identified as significant interactions. The colorectum tumor sample was obtained from Zhongnan Hospital of Wuhan University in Wuhan, China.

### eQTL analysis in our own CRC tissues

A total of 241 CRC patients were recruited from Tongji Hospital of Huazhong University of Science and Technology and Zhongnan Hospital of Wuhan University, Wuhan, China. Genomic DNA used for SNP rs140356782 genotyping was extracted from peripheral blood samples using the Relax Gene Blood DNA System Kit (Tiangen, China). The SNP rs140356782 was genotyped by the TaqMan SNP real-time polymerase chain reaction (PCR) assay (Applied Biosystems, USA). The expression of candidate gene *PANK1* was measured using qRT-PCR from tumor tissues of CRC patients. The *P* values were calculated by a two-sided Student’s *t*-test. Informed consent was obtained from each subject, and this study was approved by the Biomedical Ethics Committee of Wuhan University.

### Statistical analysis

Population distribution of genetic risk in different groups was plotted for each model to assess how well PRS differentiates samples with different disease stages. The predictive performance of each PRS model was assessed and compared by AUC calculated via ROCt. Adjusted odds ratios (ORs) and 95% confidence intervals (CIs) for each additional SD were estimated using multivariable logistic regression. Age, sex, race, family history, three principal components, and genotype platforms were adjusted in the corresponding risk assessment model. Besides, we calculated adjusted ORs across PRS deciles, with the lowest PRS decile as the reference category. To investigate the predictive effect of the PRS in screening, we further calculated sensitivity, specificity, positive predictive value (PPV), and negative predictive value (NPV) at the top 2%, 5%, or 10% of the PRS distribution as the high-risk group versus the rest of the individuals, adjusting for the covariates previously mentioned. And simultaneously, we calculated the ORs and CIs of these cut-off points to assess the discrimination capability of extreme categories of PRS.

PRS and ERS were incorporated into multivariable regression analysis for building the comprehensive model, adjusting for age, sex, race, family history, principal components, and genotype platform. Firstly, PRS and ERS were modeled as continuous variables (per 1 SD increase). Also, we converted both scores from a continuous variable to a categorical variable (tertiles) for analysis (low: bottom 20%; middle: mid 20–80%; top: top 20%). In addition, the synergy index (S), attributable proportion (AP), and relative excess risk due to interaction (RERI) were used to assess biological interactions in additive models, and interaction relative risk was used to assess the multiplicative interaction between the two risk scores. To evaluate the combined predictive effect of the two scores in the screening, we calculated the screening indicators mentioned before, at the top 2%, 5%, or 10% of the PRS distribution and top 20% of the ERS distribution as high-risk group. The cumulative incidence of colorectal neoplasm was calculated by using the Kaplan–Meier method. The incidence of neoplasm was compared among different risk subgroups using a time-dependent Cox hazards regression model adjusted for age, sex, family history, principal components, and genotype platform. Because the number of CRC cases included was much larger than advanced adenoma in UK Biobank, we performed a sensitivity analysis that cases only included only patients with advanced adenoma. All other statistical analyses were performed using the R statistical software ver.4.1.2. Two-sided *P* value of < 0.05 was considered statistically significant.

## Results

### Three trans-ancestry PRSs development and assessment

**Fig. 1:**
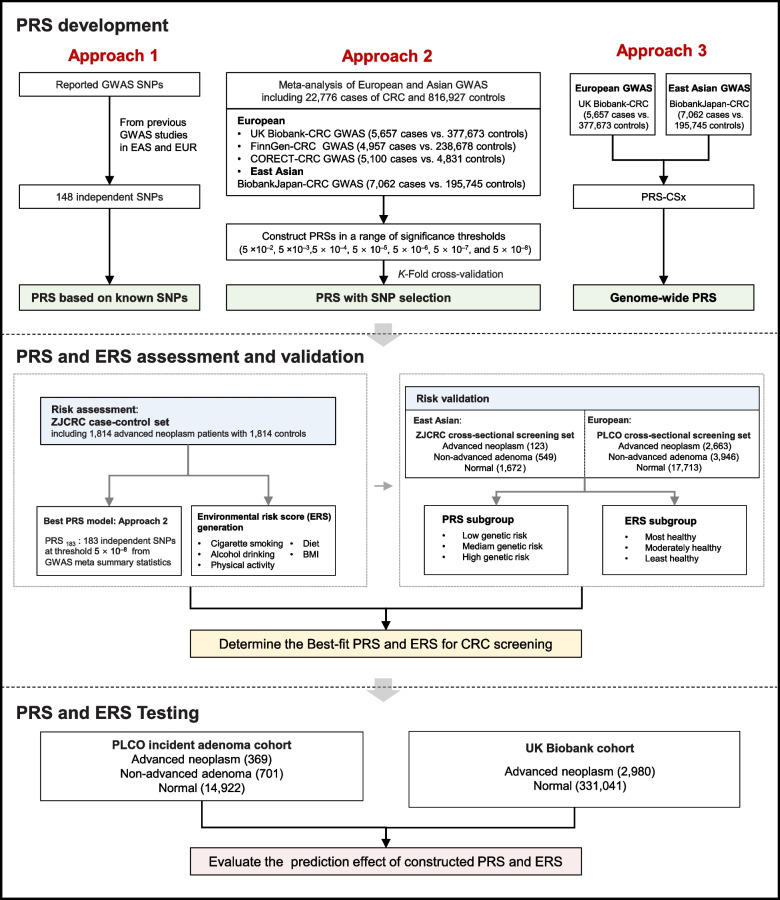
Overview of the study design. First, three trans-ancestry PRS approaches were implemented. After assessment and validation in the East Asian and European ancestry screening populations, the best-fitting PRS model together with generated ERS for CRC screening was determined. At last, the prediction effect of constructed PRS and ERS was further evaluated in cohorts

**Fig. 2:**
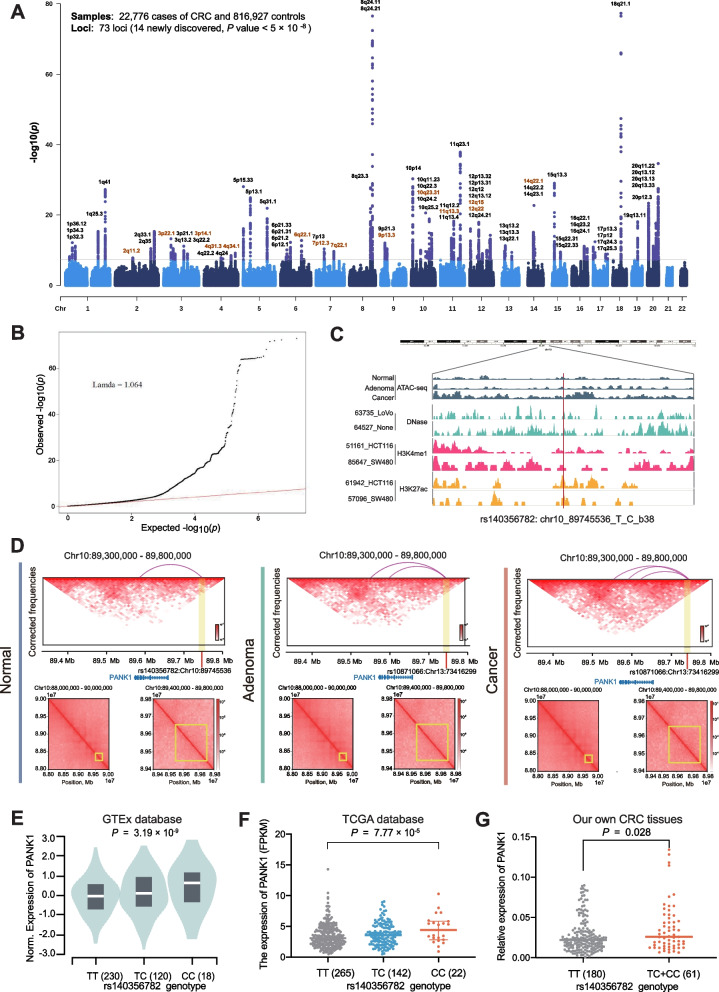
Quality control of GWAS meta-analysis and functional annotation of the newly discovered risk variant rs140356782 in 10q23.31. **A** Manhattan plot of GWAS meta-analysis results, the *P* values (-log10) of the SNPs (*y*-axis) are presented according to their chromosomal positions (*x*-axis). **B** QQ plot of GWAS meta-analysis. **C** Epigenetic tracks obtained from the ATAC-seq peaks of our own three-stage tissues (CRC, adenoma, and normal) and ENCODE database show the enrichment of enhancer marks (DNase modification peaks, H3K4me1, and H3K27ac peaks) in the rs140356782 region. **D** Hi-C plots reveal the interaction of the region containing rs140356782 with PANK1 promoter in our own three-stage tissues (CRC, adenoma, and normal). **E**–**G** eQTL analyses demonstrate the correlation between rs140356782 genotype and the expression of PANK1 in the GTEx normal transverse colon samples (**E**), TCGA colorectal cancer samples (**F**), and our own colorectal cancer tissues (**G**). Data are shown as the median (minimum to maximum). *P* values were calculated by a two-sided Student’s *t* test in own colorectal cancer tissues, respectively

To construct an optimal trans-ancestry PRS for risk stratification in CRC screening, we developed PRS models by three approaches in the ZJCRC case–control set (Fig. 1). We genotyped genome-wide variants using the ASA system in a total of 3628 individuals, matched in a 1:1 ratio from the ZJCRC case–control set (Additional file 2: Figure S2, Additional file 1: Table S7). In approach 1, the PRS was derived based on 148 independent known SNPs associated with CRC risk reported in previous research [24, 25] (named PRS_148_). In approach 2, PRSs were conducted in a range of thresholds with SNP selection by clumping and thresholding based on summary statistics from the large-scale EAS-EUR CRC GWAS meta-analysis (22,776 cases and 816,927 controls). From the meta-analysis, we identified 73 loci (183 independent SNPs, LD *r*^2^ < 0.1) associated with CRC risk at genome-wide significance level (*P* < 5 × 10^−8^; Additional file 2: Fig. 2A-B, Additional file 1: Table S10). Among the 73 loci, 14 novel risk loci were found to be independently associated with CRC risk. Especially, functional annotation showed that rs140356782 located in 10q23.31 is enriched within active histone modification peaks (H3K4me1 and H3K27ac) and open chromatin accessibility (DNase-seq and ATAC-seq peaks; Fig. 2C). We further validated that the region containing rs140356782 significantly interacted with *PANK1* promoter in our colorectal tissues spanning normal to advanced adenoma to cancer using Hi-C assay (Fig. 2D). Moreover, this variant has statistically significant eQTLs with *PANK1* expression levels in three independent datasets (Fig. 2E–G). It was hypothesized that rs140356782 may affect the occurrence of CRC by regulating the expression of *PANK1*. This suggestion that our meta-analysis, with larger sample sizes and comprehensive variant ascertainment, would better assess genetic architecture of colorectal neoplasm across diverse populations. Based on the results of this meta-analysis, it was determined that PRS_183_, which consists of 183 SNPs, outperformed other threshold-based PRS models in approach 2 (Additional file 1: Table S8). In approach 3, the PRS was derived from the whole genome by PRS-CSx, a cross-population Bayesian polygenic modeling (named PRS_Genomewide_).

**Fig. 3:**
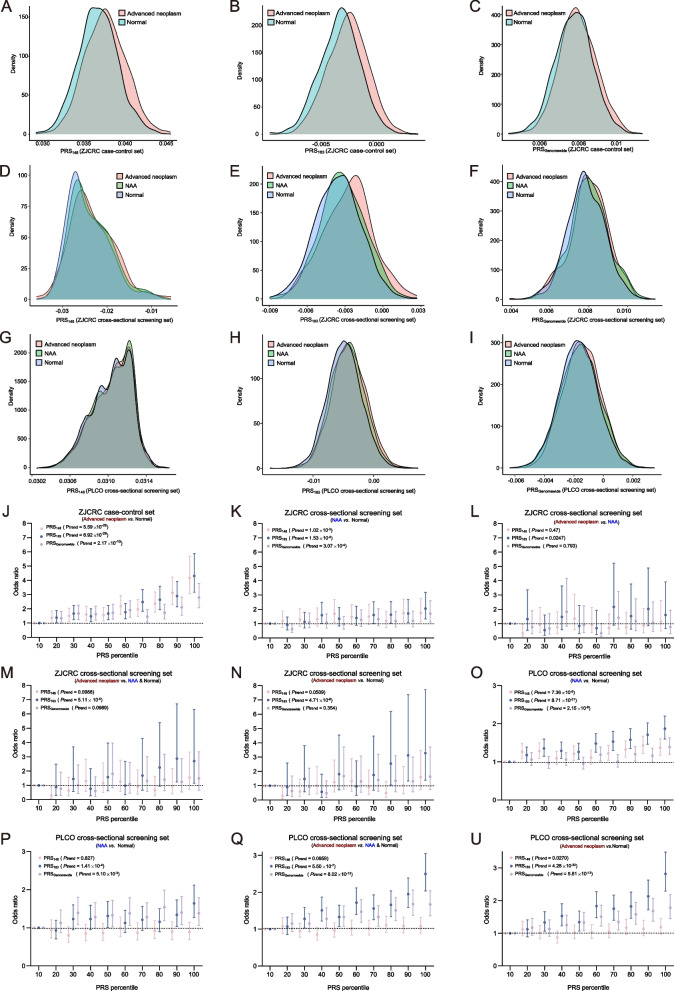
Risk score distributions and effect comparison of three approaches to PRS construction and in assessment and validation set. **A**–**C** Distribution of PRS_148_ (**A**), PRS_183_ (**B**), and PRS_Genomewide_ (**C**) in ZJCRC case–control set respectively. **D**–**F** Distribution of PRS_148_ (**D**), PRS_183_ (**E**), and PRS_Genomewide_ (**F**) in ZJCRC cross-sectional screening set respectively. **G**–**I** Distribution of PRS_148_ (**G**), PRS_183_ (**H**), and PRS_Genomewide_ (**I**) in PLCO cross-sectional screening set respectively. **J** ORs of three PRS models for each PRS decile in ZJCRC case–control set. **K**–**N** ORs of three PRS models for each PRS decile through different groups and comparisons in ZJCRC cross-sectional screening set. **O**–**U** ORs of three PRS models for each PRS decile through different groups and comparisons in PLCO cross-sectional screening set

The distribution of all three PRSs in individuals with advanced colorectal neoplasm showed a tendency towards higher values compared to the normal control group. These PRS values follow a distribution that approximated normality, and the distinction of PRS_183_ between the cases and controls is particularly pronounced (Fig. 3A–C). Notably, PRS_183_ demonstrate a superior discriminatory ability in distinguishing colorectal advanced neoplasm from normal controls compared to the other two PRSs (AUC_adjust_ = 0.607 vs. 0.604 for PRS_148_ and 0.560 for PRS_Genomewide_, Table 1). Additionally, a greater PRS value was linked to increased risk for advanced colorectal neoplasm in three models, with PRS_183_ exhibiting the most pronounced effect [OR_per sd_ = 1.48 (*P* = 3.20 × 10^−30^) vs. OR_per sd_ = 1.46(*P* = 3.90 × 10^−28^) for PRS_148_ and OR_per sd_ = 1.25(*P* = 2.42 × 10^−11^) for PRS_Genomewide_] (Table 1). We also noticed a gradient of risk across different levels of PRS, with subjects in the higher decile of the PRSs being significantly more susceptible to colorectal neoplasm. Especially, PRS_183_ exhibited the most significant dose–response effect among the three models OR_p10 vs. p1_ = 4.31, *P*_trend_ = 6.92 × 10^−29^) (Fig. 3J).

**Table 1:** The performance metrics of three PRS construction strategies in the assessment set and validation set by ancestry

Population	Cases/controls	Approach 1 (PRS_148_)		Approach 2 (PRS_183_)		Approach 3 (PRS_Genomewide_)	
		AUC (crude)	OR per s.d. (95% CI), *P*	AUC (crude)	OR per s.d. (95% CI), *P*	AUC (crude)	OR per s.d. (95% CI), *P*
**ZJCRC case–control set**							
Advanced neoplasm vs normal	1814/1814	0.604 (0.605)	1.46 (1.37–1.56),* P* = 3.90 × 10^−28^	0.607 (0.609)	1.48 (1.38–1.58), *P* = 3.20 × 10^−30^	0.560 (0.559)	1.25 (1.17–1.34), *P* = 2.42 × 10^−11^
**ZJCRC cross-sectional screening set**							
NAA vs normal	549/1672	0.536 (0.540)	1.04 (0.94–1.15),* P* = 0.415	0.556 (0.558)	1.25 (1.14–1.38),* P* = 5.87 × 10^−6^	0.550 (0.548)	1.21 (1.09–1.33),* P* = 1.92 × 10^−4^
Advanced neoplasm vs NAA	123/549	0.517 (0.505)	1.00 (0.82–1.23),* P* = 0.968	0.567 (0.555)	1.24 (1.02–1.52),* P* = 0.0305	0.504 (0.509)	1.01 (0.82–1.23),* P* = 0.932
Advanced neoplasm vs NAA and Normal	123/2221	0.543 (0.535)	1.02 (0.85–1.23),* P* = 0.796	0.586 (0.607)	1.49 (1.24–1.8),* P* = 2.14 × 10^−5^	0.547 (0.527)	1.17 (0.97–1.41),* P* = 0.978
Advanced neoplasm vs normal	123/1672	0.552 (0.546)	1.03 (0.86–1.24),* P* = 0.725	0.591 (0.620)	1.61 (1.33–1.94),* P* = 8.79 × 10^−7^	0.543 (0.557)	1.24 (1.02–1.5),* P* = 0.0276
**PLCO cross-sectional screening set**							
NAA vs normal	3946/17,713	0.515 (0.503)	0.99 (0.93–1.04),* P* = 0.639	0.546 (0.550)	1.19 (1.15–1.23),* P* = 9.20 × 10^−23^	0.525 (0.525)	1.10 (1.06–1.13),* P* = 2.40 × 10^−7^
Advanced neoplasm vs NAA	2663/3946	0.531 (0.527)	1.13 (1.07–1.2), *P* = 1.57 × 10^−5^	0.531 (0.529)	1.13 (1.07–1.2),* P* = 1.57 × 10^−5^	0.526 (0.521)	1.09 (1.03–1.15),* P* = 4.27 × 10^−3^
Advanced neoplasm vs NAA and normal	2663/21,659	0.525 (0.511)	1.00 (0.96–1.04),* P* = 0.996	0.570 (0.569)	1.30 (1.24–1.35),* P* = 9.66 × 10^−31^	0.542 (0.546)	1.17 (1.12–1.22),* P* = 2.21 × 10^−12^
Advanced neoplasm vs normal	2663/17,713	0.523 (0.515)	1.01 (0.96–1.05),* P* = 0.809	0.582 (0.577)	1.34 (1.28–1.4), *P* = 4.69 × 10^−37^	0.549 (0.546)	1.19 (1.14–1.25),* P* = 1.58 × 10^−14^

^*^The model includes age, sex, family history, principal components, genotype platform, and continuous z-transformed PRS. Advanced neoplasm, including CRC cases and advanced adenoma. *NAA* non-advanced adenoma, *OR* odd ratio, *AUC* area under curve

Subsequently, we estimated the OR for individuals in the top 2%, 5%, and 10% of the PRS compared with the remaining individuals. Apparently, individuals at the top tails of the PRSs consistently exhibited elevated risk of advanced neoplasm compared to the bottom, regardless of the specific cutoff points used. Importantly, PRS_183_ consistently demonstrated superior performance [e.g., OR_Top 5%_ = 2.50 (*P* = 3.34 × 10^−5^) of PRS_183_; OR_Top 5%_ = 2.21 (*P* = 1.51 × 10^−6^) of PRS_148_; OR_Top 5%_ = 1.76 (*P* = 3.21 × 10^−4^) of PRS_Genomewide_] (Table 2, Additional file 1: Table S17-18). In conclusion, PRS_183_ showed the highest efficacy among all the models to differentiate people at a higher risk of developing colorectal advanced neoplasm from normal people in the assessment set.

**Table 2:** Prediction accuracy of three approach of the contrasted trans-ancestry PRS (5% of the PRS distribution as classifier)

Population	PRS threshold: top 5% versus other 95%				
	OR (95% CI), *P*	Sensitivity	Specificity	PPV	NPV
**Approach 1 (PRS**_**148**_**)**					
**ZJCRC case–control set**					
Advanced neoplasm vs normal	2.21 (1.61–3.05), *P* = 1.51 × 10^−6^	-	-	-	-
**ZJCRC cross-sectional screening set**					
NAA vs normal	1.16 (0.75–1.79), *P* = 0.513	0.05	0.95	0.27	0.75
Advanced neoplasm vs NAA	1.03 (0.41–2.58), *P* = 0.945	0.05	0.95	0.18	0.82
Advanced neoplasm vs NAA and normal	0.98 (0.42–2.31), *P* = 0.971	0.05	0.95	0.05	0.95
Advanced neoplasm vs normal	0.94 (0.4–2.24), *P* = 0.897	0.05	0.95	0.07	0.93
**PLCO cross-sectional screening set**					
NAA vs normal	0.85 (0.72–1.01), *P* = 0.0595	0.04	0.95	0.16	0.82
Advanced neoplasm vs NAA	1.24 (0.99–1.55), *P* = 0.0588	0.06	0.95	0.45	0.6
Advanced neoplasm vs NAA and normal	1.09 (0.91–1.31),* P* = 0.344	0.05	0.95	0.12	0.89
Advanced neoplasm vs normal	1.04 (0.87–1.25), *P* = 0.663	0.05	0.95	0.14	0.87
**Approach 2 (PRS**_**183**_**)**					
**ZJCRC case–control set**					
Advanced neoplasm vs normal	2.50 (1.81–3.47), *P* = 3.34 × 10^−5^	-	-	-	-
**ZJCRC cross-sectional screening set**					
NAA vs normal	1.76 (1.17–2.64), *P* = 6.58 × 10^−3^	0.07	0.96	0.36	0.76
Advanced neoplasm vs NAA	1.63 (0.73–3.61), *P* = 0.23	0.07	0.95	0.26	0.82
Advanced neoplasm vs NAA and normal	2.62 (1.43–4.78), *P* = 1.77 × 10^−3^	0.11	0.95	0.12	0.95
Advanced neoplasm vs normal	2.98 (1.62–5.5), *P* = 4.64 × 10^−4^	0.12	0.96	0.17	0.94
**PLCO cross-sectional screening set**					
NAA vs normal	1.48 (1.28–1.71), *P* = 1.05 × 10^−7^	0.07	0.95	0.24	0.82
Advanced neoplasm vs NAA	1.42 (1.14–1.78), *P* = 1.82 × 10^−3^	0.06	0.96	0.48	0.6
Advanced neoplasm vs NAA and normal	1.78 (0.99–1.55), *P* = 3.88 × 10^−13^	0.08	0.95	0.17	0.89
Advanced neoplasm vs normal	1.94 (1.66–2.27), *P* = 9.88 × 10^−17^	0.08	0.95	0.21	0.87
**Approach 3 (PRS**_**Genomewide**_**)**					
**ZJCRC case–control set**					
Advanced neoplasm vs normal	1.76 (1.29–2.40),* P* = 3.21 × 10^−4^	-	-	-	-
**ZJCRC cross-sectional screening set**					
NAA vs normal	2.12 (1.42–3.17), *P* = 2.52 × 10^−4^	0.08	0.96	0.38	0.76
Advanced neoplasm vs NAA	1.05 (0.42–2.62),* P* = 0.918	0.05	0.95	0.18	0.82
Advanced neoplasm vs NAA and normal	1.28 (0.58–2.84), *P* = 0.544	0.06	0.95	0.06	0.95
Advanced neoplasm vs normal	1.63 (0.76–3.52), *P* = 0.213	0.07	0.95	0.09	0.93
**PLCO cross-sectional screening set**					
NAA vs normal	1.31 (1.13–1.52), *P* = 3.36 × 10^−4^	0.06	0.95	0.22	0.82
Advanced neoplasm vs NAA	1.42 (1.14–1.78),* P* = 1.82 × 10^−3^	0.06	0.96	0.48	0.6
Advanced neoplasm vs NAA and normal	1.23 (1.03–1.46), *P* = 0.0216	0.06	0.95	0.13	0.89
Advanced neoplasm vs normal	1.30 (1.09–1.54), *P* = 3.41 × 10^−3^	0.06	0.95	0.16	0.87

^*^Models were adjusted for age, sex, family history, genotype platform, and principal components. Two-sided *P* values per the Wald test. The error bars represent the 95% confidence intervals (CIs). *NAA* non-advanced adenoma, *OR* odd ratio, *PPV* positive predictive value, *NPV* negative predictive value

### Optimal PRS-PRS_183_ showed strong performance in EAS and EUR validation

To validated the PRS models across all stages of the dynamic adenoma-carcinoma sequence, we evaluated their performance in cross-sectional screening datasets from our East Asian cohort and replicated in an independent European population cohort. We applied a high-throughput ASA chip to gain genome-wide variants information in the ZJCRC (EAS) cross-sectional screening datasets (Additional file 2: Figure S1, Additional file 1: Table S7). We observed that the distribution of each PRS follows a pattern of increasing trend from normal, non-advanced adenoma, to advanced neoplasm (Fig. 3D–I). This trend suggested that the PRSs had the potential to be utilized extensively as a risk assessment tools for all stages of colorectal neoplasm progression. Likewise, PRS_183_ exhibited the best precise discrimination ability in both EAS and EUR population.

To further explore whether such properties of PRSs can be exploited well for discriminating distinct malignant stage, we employed four distinct ways of comparison based on the progressive stages of the carcinogenic process (Table 1). It is noteworthy that the PRS_183_ models demonstrate robust predictive performance in distinguishing advanced neoplasms from normal in both East Asian (AUC_adjusted_ = 0.591) and European (AUC_adjusted_ = 0.582) populations respectively (Table 1). In addition, it was possible to differentiate the advanced neoplasm group from the NAA group to a certain extent as well (e.g., AUC_adjusted_ of PRS_183_ = 0.567 in the ZJCRC cross-sectional screening set, and 0.531 in the PLCO cross-sectional screening set) (Table 1). Interestingly, PRS_183_ also had discriminative capacity when comparing the NAA group to the normal group (Table 1).

Compared to the other PRS models, PRS_183_ demonstrated a stronger correlation with the progression of neoplasm across different stages (Table 1). In the ZJCRC cross-sectional screening set, a continuous rise in PRS_183_ was associated with an increased risk of advanced neoplasms, whether compared to the normal control group (OR = 1.61), the NAA control group (OR = 1.24), or both as control group (OR = 1.49) (Table 1). Moreover, PRS_183_ was similarly linked to an increased risk of developing NAA (OR = 1.25) (Table 1). Intriguingly, similar trends were found in PLCO cross-sectional screening set, further demonstrating the improved transferability of PRS_183_ in non-EUR populations (Table 1). As expected, PRS_183_ still demonstrated the most significant trend and effect among the three models across PRS deciles in different case–control groups in both EAS and EUR sets (Fig. 3K–U). To assess the utility of the PRS, we found that the positive predictive values (PPV) at the top 5% PRS183 for NAA and advanced neoplasm ranged from 0.12 to 0.36, and the negative predictive value (NPV) ranged from 0.76 to 0.95 indicated by colonoscopy in the ZJCRC screening set (Table 2). It is crucial to note that sensitivity values are influenced by the chosen threshold for defining high-risk groups. Specifically, using a higher percentage (top 5%) as the classifier tends to result in higher sensitivity compared to a lower percentage (top 2%) (Table 2, Additional file 1: Table S17-18) [29]. These findings indicated that PRS_183_ showed a more pronounced accuracy in positive and negative identification of colorectal neoplasm, especially in advance colorectal neoplasm. Considering the overall performance in the assessment and validation sets, PRS_183_ demonstrated superior performance compared to the other two PRS models, offering thorough risk assessment across all stages of the dynamic adenoma-carcinoma sequence in both the EAS and EUR populations. As a result, it was selected as the optimal PRS for further analyses.

### Environmental risk scores (ERS) generation and evaluation

As the unfavorable modifiable lifestyle factors (including cigarette smoking, alcohol drinking, less physical activity, unhealthy diet, and high body weight) showed increased risk of colorectal advanced neoplasm, we constructed the ERS with these factors subsequently (Additional file 1: Table S11). In the ZJCRC case–control set, a higher ERS was found to be associated with an increased risk of advanced neoplasm (OR_per sd_ = 1.35, *P* = 1.22 × 10^−18^), indicating a dose–response relationship (Additional file 1: Table S19). In both validation datasets, the performance of ERS was not very impressive when comparing the advanced neoplasm group with normal individuals. However, ERS exhibited relatively better predictive performance (AUC_adjust_ = 0.553 in the ZJCRC cross-sectional screening set; AUC_adjust_ = 0.543 in the PLCO cross-sectional screening set) when comparing the NAA group with normal individuals (Additional file 1: Table S19).

**Fig. 4:**
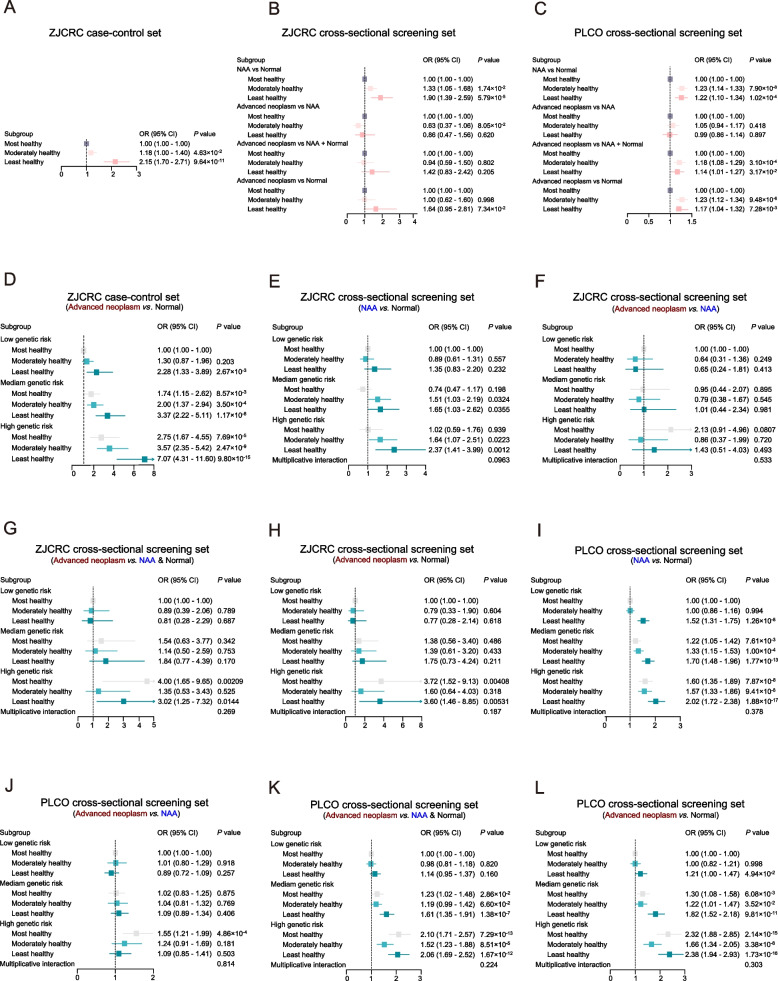
The risk of colorectal cancer screening according to PRS and ERS categories across three assessment and validation set. **A** ORs for colorectal neoplasms in low, intermediate, and high environmental risk groups in the ZJCRC case–control set. **B** ORs for colorectal neoplasms in low, intermediate, and high environmental risk groups through different groups and comparisons in ZJCRC cross-sectional screening set. **C** ORs for colorectal neoplasms in low, intermediate, and high environmental risk groups through different groups and comparisons in PLCO cross-sectional screening set. **D** ORs for colorectal neoplasms according to genetic and environmental categories in the ZJCRC case–control set. **E**–**H** ORs for colorectal neoplasms according to genetic and environmental categories through different groups and comparisons in ZJCRC cross-sectional screening set. **I**–**L** ORs for colorectal neoplasms according to genetic and environmental categories through different groups and comparisons in PLCO cross-sectional screening set. ORs are adjusted for age, sex, family history, principal components, and genotype platform. 95% confidence intervals are shown for all analyses

Furthermore, we discovered that there was an incremental risk at stratified level in addition to the continuous level. Contrasted with the most healthy group, the least healthy group had a 2.15 times higher risk of advanced neoplasm (*P* = 9.64 × 10^−11^), and the moderately healthy group had 1.18 times higher risk (*P* = 4.63 × 10^−2^) (Fig. 4A). When comparing the advanced neoplasm group to the normal group (top 20% ERS as a classifier), the PPV and NPV were estimated to be 0.13 and 0.94 in the ZJCRC cross-sectional screening set and 0.14 and 0.87 in the PLCO cross-sectional screening set (Table 3). However, although the overall trend remained the same, ERS exhibited relatively poor discriminant performance in the validation set (Fig. 4B, C).

**Table 3:** Prediction accuracy of ERS and PRS-ERS strategy

Population	ERS threshold: top 20% versus other 80%					PRS-ERS threshold: ERS top 20% and PRS top 5% versus other				
	OR/HR (95% CI), *P*	Sensitivity	Specificity	PPV	NPV	OR/HR (95% CI), *P*	Sensitivity	Specificity	PPV	NPV
**ZJCRC case–control set**										
Advanced neoplasm vs normal	1.87 (1.55–2.26),* P* = 5.59 × 10^−11^	-	-	-	-	5.00 (2.21–11.34),* P* = 1.14 × 10^−4^	-	-	-	-
**ZJCRC cross-sectional screening set**										
NAA vs normal	1.57 (1.20–2.05),* P* = 9.94 × 10^−4^	0.27	0.84	0.35	0.78	2.05 (0.96–4.41),* P* = 0.0649	0.02	0.99	0.46	0.76
Advanced neoplasm vs NAA	1.15 (0.69–1.91),* P* = 0.598	0.24	0.81	0.22	0.83	3.58 (0.93–13.74),* P* = 0.0631	0.03	0.99	0.44	0.82
Advanced neoplasm vs NAA and normal	1.47 (0.94–2.3),* P* = 0.0894	0.33	0.81	0.09	0.96	4.05 (1.66–9.88),* P* = 2.08 × 10^−3^	0.06	0.99	0.23	0.95
Advanced neoplasm vs normal	1.64 (1.04–2.57),* P* = 0.0317	0.34	0.82	0.13	0.94	4.51 (1.73–11.76),* P* = 2.10 × 10^−3^	0.06	0.99	0.32	0.93
**PLCO cross-sectional screening set**										
NAA vs normal	1.10 (1.00–1.20),* P* = 0.0482	0.18	0.83	0.19	0.82	1.85 (1.34–2.55),* P* = 1.73 × 10^−4^	0.01	0.99	0.29	0.82
Advanced neoplasm vs NAA	0.97 (0.85–1.10),* P* = 0.635	0.18	0.82	0.4	0.6	1.60 (0.93–2.75),* P* = 0.0888	0.01	0.99	0.49	0.6
Advanced neoplasm vs NAA and normal	1.05 (0.94–1.16),* P* = 0.411	0.18	0.83	0.11	0.89	2.38 (1.62–3.5),* P* = 9.66 × 10^−6^	0.02	0.99	0.19	0.89
Advanced neoplasm vs normal	1.06 (0.95–1.18),* P* = 0.281	0.18	0.83	0.14	0.87	2.61 (1.76–3.88),* P* = 2.12 × 10^−6^	0.02	0.99	0.25	0.87
**PLCO incident adenoma cohort**										
NAA vs normal	1.04 (0.85–1.26),* P* = 0.718	0.17	0.84	0.05	0.96	2.06 (1.14–3.74), *P* = 0.0172	0.02	0.99	0.09	0.96
Advanced neoplasm vs normal	1.14 (0.92–1.41), *P* = 0.231	0.16	0.84	0.02	0.98	1.76 (0.73–4.27), *P* = 0.207	0.01	0.99	0.04	0.98
**UK Biobank cohort**										
Advanced neoplasm vs normal	1.22 (0.97–1.55), *P* = 0.0901	0.48	0.62	0.01	0.99	4.49 (2.65–7.59), *P* = 2.11 × 10^−8^	0.02	0.98	0.04	0.98

^*^Models were adjusted for age, sex, family history, genotype platform, and principal components. Two-sided *P* values per the Wald test. The error bars represent the 95% confidence intervals (CIs). Advanced neoplasm, including CRC cases and advanced adenoma. *NAA* non-advanced adenoma, *OR* odds ratio, *HR* hazard ratio, *PPV* positive predictive value, *NPV* negative predictive value

### The incorporation of PRS and ERS in risk prediction

To integrate a more comprehensive strategy and provide a more complementary risk assessment for screening, we further investigated the association between the incorporating effects of PRS and ERS in different colorectal progressive stages. We indicated the overall increasing risk trend with increasing comprehensive risk score across different datasets and different comparison ways (Fig. 4D–L, Additional file 2: Figure S3). This trend is most evident in the ZJCRC case–control set, where individuals in the least healthy and highest risk category had a 7.07 times higher risk (*P* = 9.80 × 10^−15^) compared to those in the lowest category (Fig. 4D). Besides, integrating both PRS and ERS into a combined model improved discrimination compared to PRS or ERS alone (e.g., AUC = 0.629 vs. 0.607/0.581 in the ZJCRC case–control set) (Additional file 1: Table S19).

Moreover, in the extreme right tail of both PRS and ERS distribution, a notable increase in the risk of different stages of colorectal neoplasm and malignant potential was observed (Table 3, Additional file 1: Table S21, S22). These findings suggest that the incorporation of PRS and ERS can further optimize the screening strategy for individuals at high risk of colorectal neoplasms. When individuals in the both top 5% of PRS_183_ and top 20% ERS were defined as high-risk group, the PPV and NPV was improved for detecting advanced neoplasm compared to PRS or ERS alone (PPV = 0.32, NPV = 0.93 in ZJCRC cross-sectional screening set; PPV = 0.25, NPV = 0.87 in PLCO cross-sectional screening set) (Table 3). Additionally, there was evidence of moderately positive additive interactions between genetic and environmental risk factors (e.g., 0.31 [0.06, 0.58], AP = 0.13 [0.02, 0.23], S = 1.31 [1.05, 1.65] in ZJCRC case–control set) (Additional file 1: Table S20).

### The comprehensive model superior to PRS and ERS alone in testing cohort

**Fig. 5:**
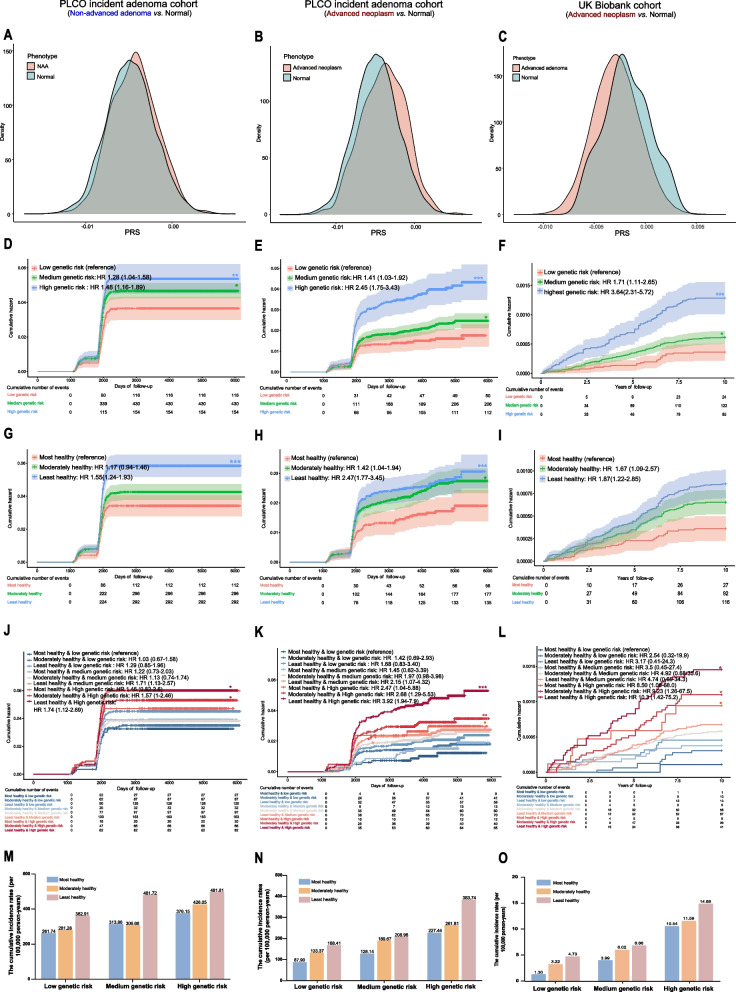
Evaluation of absolute risk predictions of incident colorectal neoplasm according to PRS and ERS in the PLCO and UK Biobank cohort. **A** Distribution of PRS when non-advanced adenoma vs normal group within PLCO incident adenoma cohort. **B**, **C** Distribution of PRS when advanced adenoma vs normal group within PLCO incident adenoma cohort (**B**) and UK Biobank cohort (**C**). **D** Inverted Kaplan–Meier plot of incident colorectal neoplasm by PRS when non-advanced neoplasm vs normal group within PLCO incident adenoma cohort. **E**, **F** Inverted Kaplan–Meier plot of incident colorectal neoplasm by PRS when advanced neoplasm vs normal group within PLCO incident adenoma cohort (**E**) and UK Biobank cohort (**F**). **G** Inverted Kaplan–Meier plot of incident colorectal neoplasm by ERS when non-advanced adenoma vs normal group within PLCO incident adenoma cohort. **H**, **I** Inverted Kaplan–Meier plot of incident colorectal neoplasm by ERS when advanced neoplasm vs normal group within PLCO incident adenoma cohort (**H**) and UK Biobank cohort (**I**). Participants were divided into most, moderately, and least healthy groups. **J** Inverted Kaplan–Meier plot of incident colorectal neoplasm according to genetic and environmental categories when non-advanced adenoma vs normal group within PLCO incident adenoma cohort. **K**–**L** Inverted Kaplan–Meier plot of incident colorectal neoplasm according to genetic and environmental categories when advanced neoplasm vs normal group within PLCO incident adenoma cohort (**K**) and UK Biobank cohort (**L**). Participants were divided into 9 risk groups. The cumulative events table under the plot showed the cumulative incident events of incident colorectal neoplasm cases at years of follow-up. **M** Per 100,000 person-year at risk separately in 9 risk groups in the context of non-advanced adenoma vs normal group within PLCO incident adenoma cohort. **N**, **O** Per 100,000 person-year at risk separately in 9 risk groups in the context of advanced neoplasm vs normal group within PLCO incident adenoma cohort (**N**) and UK Biobank cohort (**O**)

In the testing phase, we performed additional examinations to evaluate the predictive capabilities of PRS_183_ and ERS in two prospective cohorts, namely the PLCO incident adenoma cohort and the UK Biobank cohort. We proved that PRS_183_ can be effectively used in screening for high-risk populations of colorectal neoplasm in screening cohorts (Fig. 5A–C). Strikingly, the incorporation of both PRS and ERS into a comprehensive strategy enhances risk stratification and improves discriminatory accuracy when compared to the application of PRS and ERS alone (AUC_adjusted_ = 0.602 in the PLCO incident adenoma cohort; AUC_adjusted_ = 0.630 in the UK Biobank cohort) (Table 2). Similarly, there were notable disparities in the risk of incidents among various PRS and ERS groups during the follow-up period (Fig. 5D–I). More importantly, among the subgroups, individuals in the subgroup characterized by the least health and high genetic risk exhibited the highest risk of developing colorectal neoplasms: the absolute risk of non-advanced adenoma in the subgroup was calculated to be 1.74 (*P* = 0.0133) in the PLCO incident adenoma cohort; the absolute risk of advanced neoplasm was 3.92 (*P* = 1.35 × 10^−4^) in the PLCO incident adenoma cohort and 4.31 (*P* = 4.55 × 10^−15^) in the UK Biobank cohort (Fig. 5J–L, Additional file 2: Figure S4). Likewise, the cumulative incidence rate per person-year also exhibited the highest in the subgroup (Fig. 5M–O). Sensitivity analysis yielded consistent findings, as depicted in Additional file 2: Figure S5. In brief, our comprehensive risk-stratified model employed in population-based CRC screening trails represents a promising advance in personalized risk assessment.

## Discussion

Timely intervention for high-risk individuals through the early detection of colorectal neoplasms can mitigate the burden of CRC. Nevertheless, the East Asian population lacks effective risk prediction models for personalized CRC screening. Leveraging large-scale independent multiethnic GWASs data from screening sets, we provided a framework for optimizing a trans-ancestry PRS for colorectal neoplasms. The best-fitting model, PRS_183_, exhibits strong predictive performance and effective identification of individuals at high risk of advanced colorectal neoplasm across diverse ancestry populations, particularly within EAS population. Notably, incorporating the lifestyle factors into the comprehensive risk-stratified strategy achieves improved predictive performance compared to the PRS or ERS individually. Overall, our model represents a promising advancement in personalized risk assessment, promoting PRS transferability across ancestries to tackle health disparities.

Currently, challenges arise from the decreased trans-ancestry prediction accuracy of PRS, especially when the discovery and target populations are genetically distant. While various PRS models have been developed to enhance the predictive ability of complex diseases, these traditional models have primarily focused on specific ethnic populations, exemplified by methods like P + T [30], PRS-CS [31], and LDpred [32], which typically require larger GWAS sample sizes to achieve higher prediction accuracy. As existing GWASs were predominantly conducted in European populations [33, 34], the poor transferability of PRS across populations has impeded its clinical implementation and raised health disparity concerns. To tackle this challenge, many studies have proposed methodological improvements to address the limitations of traditional PRS models, including PolyPred [35], PRS-CSx [15], and XPASS [16], which might improve the trans-ancestry polygenic prediction through reducing heterogeneity or sharing information between ancestries. Expectedly, three trans-ancestry PRSs in our study achieved moderately improved predictive performance in EAS compared to EUR populations. The noteworthy improvement in PRS_183_ is likely attributable to the utilization of a meta-analysis of the largest CRC multi-ancestry GWASs, as compared with PRS_148_ and PRS_Genomewide_. Indeed, advanced statistical and computational methods alone cannot address Eurocentric biases in GWAS. Broadening the sample diversity is essential to understand genetic architecture and non-genetic factors across global populations, enhancing PRS prediction accuracy [36].

Early detection of adenomas and subsequent radical removal is essential for breaking the adenoma-carcinoma sequence, ultimately reducing the burden of colorectal cancer. Previous studies indicated that the majority of common variants associated with CRC risk are also involved in early carcinogenesis for the adenoma-carcinoma sequence [37, 38], which could aid to refine risk stratification in screening programs. However, little is known about the effectiveness of PRS in predicting the CRC precursors in a true screening setting. Obón-Santacana et al. developed a PRS model using 133 CRC-associated SNPs to predict colorectal neoplasm in European population, with an AUC of 0.58 for advanced neoplasms and 0.56 for all neoplasms [11]. While this study proposed the importance of PRS in the CRC tumorigenesis sequence, its predictive effectiveness is limited due to the relatively small sample size and the absence of independent validation analysis. In our study, to establish a thorough and credible risk assessment across all stages of the dynamic adenoma-carcinoma sequence, we extensively covered a broad spectrum of outcomes within independent high-quality endoscopy screening sets. Interestingly, we found that the distribution of each PRS follows a pattern of increasing trend from normal, non-advanced adenoma, to advanced neoplasm. Importantly, the PRSs demonstrated superior discrimination capacity for advanced neoplasm (AUC = 0.627 for PRS_183_) and also showed some degree of differentiation between advanced neoplasm and non-advanced adenoma. These findings provide additional support for the utility of PRS as a promising stratification tool in the screening and detection of early-stage colorectal neoplasms, even among those with moderate genetic risk, who are more likely to have non-advanced adenomas. While the observed slight effect in identifying non-advanced adenomas from normal cases might indicate a room for improvement, the fact that PRS models show any effect in this area is still promising.

In addition to the genetic variants, the contribution of lifestyle factors in the development of colorectal neoplasms is gaining recognition. Multiple studies suggested that including well-established lifestyle-related CRC risk factors in risk score could lead to improved prediction accuracy [10, 39]. For example, using large case–control European populations, a model that combined 19 environmental factors and 63 SNPs enhance the discriminatory ability to CRC (AUC = 0.63, PRS model: AUC = 0.59, ERS model: AUC = 0.60) [40]. Similarly, a model comprising 141 common variants and 16 environmental factors for early-onset CRC has the potential to improve the discriminatory capacity to 0.631 (PRS model: AUC = 0.628, ERS model: AUC = 0.536) [10]. Nevertheless, there remains an insufficient understanding regarding their joint effects in predicting colorectal neoplasm. Larger studies on colonoscopy screening with detailed lifestyle information are needed in EAS populations. Strikingly, our comprehensive prediction model achieved improved predictive performance than PRS and the ERS alone across all stage of the dynamic adenoma-carcinoma sequence within EAS populations, which further improved the accuracy for early detection of CRC precursors.

Risk-stratified screening necessitates assessing the risk across all populations, incurring additional costs. Whether these added costs can be offset by potential gains in quality-adjusted life-years (QALYs) saved is a critical issue determining the feasibility of PRS application in the population. Studies propose that PRSs could enhance the efficiency of existing cancer screening programs in the UK [41]. In Chinese cancer screening programs, a PRS-stratified screening strategy modestly improves clinical benefits and cost-effectiveness [42]. As costs associated with whole-genome sequencing continue to decline, wider adoption of PRS-stratified screening may become feasible. Advances in GWAS-related technologies and mathematical approaches can enhance PRS predictiveness by incorporating newly discovered SNPs. Moreover, combining PRS with other risk factors to stratify the population into multiple risk strata may further improve the cost-effectiveness of cancer screening. In the future, conducting more precise cost calculations based on disease models and real-world data will further enhance the health economic evaluation of colorectal cancer screening.

There are still some limitations in this study. Firstly, the sample size and diversity of non-advanced adenoma cases in our cohort may be limited, affecting our model’s ability to accurately assess genetic risk at this colorectal cancer stage. To address this, we aim to expand our colorectal cancer screening cohort across all stages, focusing on increasing non-advanced adenoma samples. Additionally, improving sensitivity for early CRC detection is crucial. We plan to achieve this by incorporating more genetic markers and integrating additional biomarkers like functional SNPs and protein markers into our risk assessment model.

## Conclusions

In summary, we developed, validated, and optimized a robust trans-ancestry PRS model targeting all stages of the dynamic adenoma-carcinoma sequence in screening. By integrating PRS with modifiable lifestyle factors, we established a comprehensive risk-stratified approach to guide screening efforts. Our results hold considerable significance in improving the effectiveness of CRC screening in non-European populations, thus contributing to reducing public health disparities and offering a new insight for precise screening strategies.

### Supplementary Information

Additional file 1: Supplementary Tables. Table S1. Summary of case-control/cohort study datasets used for PRS assessment and validation. Table S2. Basic characteristics of the 1:1 matched ZJCRC case-control set. Table S3. Basic characteristics of the ZJCRC cross-sectional screening set. Table S4. Basic characteristics of the PLCO cross-sectional screening set. Table S5. Basic characteristics of the PLCO incident adenoma cohort. Table S6. Basic characteristics of the UK Biobank cohort. Table S7. Imputation quality control information. Table S8. The discriminatory accuracy of SNPs filtered by different P thresholds and LD using 400 times 5-fold cross-validation. Table S9. Summary of 148 CRC reported GWAS SNPs in European and East Asian population. Table S10. Summary of 183 selected SNPs of European and East Asian CRC GWAS meta. Table S11. The weights of environmental factors. Table S12. Summary of lifestyle factors of the ZJCRC case-control set. Table S13. Summary of lifestyle factors of the ZJCRC cross-sectional screening set. Table S14. Summary of lifestyle factors of the PLCO cross-sectional screening set. Table S15. Summary of lifestyle factors of the PLCO incident adenoma cohort. Table S16. Summary of lifestyle factors of the PLCO incident adenoma cohort. Table S17. Prediction accuracy of three approach of the contrasted trans-ancestry PRS (2% of the PRS distribution as classifier). Table S18. Prediction accuracy of three approach of the contrasted trans-ancestry PRS (10% of the PRS distribution as classifier). Table S19. Odds ratio and covariate-adjusted AUC of ERS and PRS. Table S20. The additive interaction of PRS and ERS in assessment and validation set. Table S21. Prediction accuracy of ERS and PRS-ERS strategy (20% of the ERS distribution and 2% of the PRS distribution as classifier). Table S22. Prediction accuracy of ERS and PRS-ERS strategy (20% of the ERS distribution and 10% of the PRS distribution as classifier).

Additional file 2: Supplementary Figures. Figure S1. Quality control of the genotype data of ZJCRC case-control set and ZJCRC cross-sectional screening set. Figure S2. Principal component analysis (PCA) of individuals in the assessment set and validation set and 1000 Genomes Project. Figure S3. The risk of colorectal cancer screening according to PRS&ERS categories across three assessment and validation set. Figure S4. Incident risk of colorectal neoplasm according to PRS and ERS in PLCO and UK Biobank cohort. Figure S5. Evaluation of absolute risk predictions of advanced adenoma according to PRS and ERS in UK Biobank cohort.

## Data Availability

The source data for the findings of this study are available as follows. The PLCO and UK Biobank data are publicly available upon successful application from the PLCO project (https://cdas.cancer.gov/plco/) (43) and UK Biobank (https://www.ukbiobank.ac.uk/) (44). Genotype data for CORECT have been deposited in the database of Genotypes and Phenotypes (dbGaP) under accession numbers phs001856 and phs001499 (https://www.ncbi.nlm.nih.gov/gap) (45). The GWAS summary data of BBJ (https://biobankjp.org/) and FinnGen (https://www.finngen.fi/en) (27) are available publicly online. The genotype data from the Chinese population are available on the database of the Genome Variation Map (https://bigd.big.ac.cn/gvm/getProjectDetail?Project=GVM000712). PGS weights are available via the PGS catalog (https://www.pgscatalog.org, publication ID: PGP000643, scores IDs: PGS004912).

## Abbreviations

CRC: Colorectal cancer
GWAS: Genome-wide association study
SNP: Single-nucleotide polymorphism
PRS: Polygenic risk scores
ERS: Environmental risk scores
EAS: East Asian
EUR: European
AUC: Area under curve
NPV: Negative predictive value
PPV: Positive predictive value
PLCO: The Prostate, Lung, Colorectal, and Ovarian Cancer Screening Trial
ZJCRC: Zhejiang colorectal cancer screening program
NAA: Non-advanced adenoma
OR: Odd ratio
HR: Hazard ratio
