# Supplementary material for: Developing an optimal stratification model for colorectal cancer screening and reducing racial disparities in multi-center population-based studies
Source: Genome Med. 2024 Jun 13;16:81. doi: 10.1186/s13073-024-01355-y (PMC11170922; doi:10.1186/s13073-024-01355-y)
Supplement: Supplementary file 1 — Additional file 1: Supplementary Tables. Table S1. Summary of case-control/cohort study datasets used for PRS assessment and validation. Table S2. Basic characteristics of the 1:1 matched ZJCRC case-control set. Table S3. Basic characteristics of the ZJCRC cross-sectional screening set. Table S4. Basic characteristics of the PLCO cross-sectional screening set. Table S5. Basic characteristics of the PLCO incident adenoma cohort. Table S6. Basic characteristics of the UK Biobank cohort. Table S7. Imputation quality control information. Table S8. The discriminatory accuracy of SNPs filtered by different P thresholds and LD using 400 times 5-fold cross-validation. Table S9. Summary of 148 CRC reported GWAS SNPs in European and East Asian population. Table S10. Summary of 183 selected SNPs of European and East Asian CRC GWAS meta. Table S11. The weights of environmental factors. Table S12. Summary of lifestyle factors of the ZJCRC case-control set. Table S13. Summary of lifestyle factors of the ZJCRC cross-sectional screening set. Table S14. Summary of lifestyle factors of the PLCO cross-sectional screening set. Table S15. Summary of lifestyle factors of the PLCO incident adenoma cohort. Table S16. Summary of lifestyle factors of the PLCO incident adenoma cohort. Table S17. Prediction accuracy of three approach of the contrasted trans-ancestry PRS (2% of the PRS distribution as classifier). Table S18. Prediction accuracy of three approach of the contrasted trans-ancestry PRS (10% of the PRS distribution as classifier). Table S19. Odds ratio and covariate-adjusted AUC of ERS and PRS. Table S20. The additive interaction of PRS and ERS in assessment and validation set. Table S21. Prediction accuracy of ERS and PRS-ERS strategy (20% of the ERS distribution and 2% of the PRS distribution as classifier). Table S22. Prediction accuracy of ERS and PRS-ERS strategy (20% of the ERS distribution and 10% of the PRS distribution as classifier). [file 13073_2024_1355_MOESM1_ESM.docx]

**Developing an Optimal Stratification Model for Colorectal Cancer Screening and Reducing Racial Disparities in Multi-center Population-based Studies**

**Additional file 1:**

**Supplementary Tables.**

Table S1. Summary of case-control/cohort study datasets used for PRS assessment and validation

Table S2. Basic characteristics of the 1:1 matched ZJCRC case-control set

Table S3. Basic characteristics of the ZJCRC cross-sectional screening set

Table S4. Basic characteristics of the PLCO cross-sectional screening set

Table S5. Basic characteristics of the PLCO incident adenoma cohort

Table S6. Basic characteristics of the UK Biobank cohort

Table S7. Imputation quality control information

Table S8. The discriminatory accuracy of SNPs filtered by different P thresholds and LD using 400 times 5-fold cross-validation

Table S9. Summary of 148 CRC reported GWAS SNPs in European and East Asian population

Table S10. Summary of 183 selected SNPs of European and East Asian CRC GWAS meta

Table S11. The weights of environmental factors

Table S12. Summary of lifestyle factors of the ZJCRC case-control set

Table S13. Summary of lifestyle factors of the ZJCRC cross-sectional screening set

Table S14. Summary of lifestyle factors of the PLCO cross-sectional screening set

Table S15. Summary of lifestyle factors of the PLCO incident adenoma cohort

Table S16. Summary of lifestyle factors of the PLCO incident adenoma cohort

Table S17. Prediction accuracy of three approach of the contrasted trans-ancestry PRS (2% of the PRS distribution as classifier)

Table S18. Prediction accuracy of three approach of the contrasted trans-ancestry PRS (10% of the PRS distribution as classifier).

Table S19. Odds ratio and covariate-adjusted AUC of ERS and PRS

Table S20. The additive interaction of PRS and ERS in assessment and validation set

Table S21. Prediction accuracy of ERS and PRS-ERS strategy (20% of the ERS distribution and 2% of the PRS distribution as classifier)

Table S21. Prediction accuracy of ERS and PRS-ERS strategy (20% of the ERS distribution and 10% of the PRS distribution as classifier).

| **Table S1. Summary of case-control/cohort study datasets used for PRS assessment and validation** | | | | |
| --- | --- | --- | --- | --- |
| Case-control/Cohort population | Ancestry | Status | Female (%) | Mean age (years) |
| **PRS assessment** |  |  |  |  |
| ZJCRC case-control set | East Asian ancestry | Normal (n=1,814) | 582 (32.1) | 62.0 |
| (n=3,628) |  | Advanced neoplasm (n=1,814) | 583 (32.1) | 62.1 |
| **PRS validation** |  |  |  |  |
| ZJCRC cross-sectional screening set | East Asian ancestry | Normal (n=1,672) | 969 (58.0) | 57.0 |
| (n=2,344) |  | Non-advanced adenoma (n=549) | 241 (43.9) | 60.0 |
|  |  | Advanced neoplasm (n=123) | 39 (31.7) | 62.0 |
| PLCO cross-sectional screening set | European ancestry | Normal (n=17,713) | 8,399 (47.4) | 61.9 |
| (n=24,322) |  | Non-advanced adenoma (n=3,946) | 1,434 (36.3) | 62.2 |
|  |  | Advanced neoplasm (n=2,663) | 967 (36.3) | 63.0 |
| **PRS test** |  |  |  |  |
| PLCO incident adenoma cohort | European ancestry | Normal (n=14,922) | 7,881 (52.8) | 61.8 |
| (n=15,992) |  | Non-advanced adenoma (n=701) | 437 (62.3) | 61.3 |
|  |  | Advanced neoplasm (n=369) | 232 (62.9) | 63.2 |
| UK Biobank cohort | European ancestry | Normal (n=331,041) | 184,559 (55.8) | 55.7 |
| (n=334,021) |  | Advanced neoplasm (n=2,980) | 1,256 (42.2) | 60.8 |
| *Advanced neoplasm (CRC cases and advanced adenoma) | | | | |

| **Table S2. Basic characteristics of the 1:1 matched ZJCRC case-control set** | | | | |
| --- | --- | --- | --- | --- |
| Variables |  | All participants | Normal | Advanced neoplasm |
| N |  | 3,628 | 1,814 | 1,814 |
| Sex (%) | Female | 1,165 (32.11) | 5,82 (32.08) | 5,83 (32.14) |
|  | Male | 2,463 (67.89) | 1,232 (67.92) | 1,231 (67.86) |
| Age (year), mean ± SD |  | 62.05 (7.446) | 61.99 (7.437) | 62.12 (7.457) |
| BMI (kg/m3), mean ± SD | | 24.42 (15.750) | 23.59 (3.111) | 25.24 (22.028) |
| Smoking (%) | No | 2,083 (57.41) | 1,125 (62.02) | 958 (52.81) |
|  | Yes | 1,545 (42.59) | 689 (37.98) | 856 (47.19) |
| Alcohol drinking (%) | No | 2,470 (68.08) | 1,329 (73.26) | 1,141 (62.90) |
|  | Yes | 1,158 (31.92) | 485 (26.74) | 673 (37.10) |
| * Advanced neoplasm (CRC cases and advanced adenoma); SD, Standard Deviation | | | | |

| **Table S3. Basic characteristics of the ZJCRC cross-sectional screening set** | | | | | |
| --- | --- | --- | --- | --- | --- |
| Variables |  | All participants | Normal | Non-advanced adenoma | Advanced neoplasm |
| N |  | 2,344 | 1,672 | 549 | 123 |
| Sex (%) | Female | 1,249 (53.28) | 969 (57.95) | 241 (43.90) | 39 (31.71) |
|  | Male | 1,095 (46.72) | 703 (42.05) | 308 (56.10) | 84 (68.29) |
| Age (year), mean ± SD | | 57.59 (8.47) | 56.83 (8.58) | 59.15 (7.93) | 61.02 (7.53) |
| BMI (kg/m3), mean ± SD | | 34.05 (488.20) | 38.24 (578.26) | 23.77 (3.17) | 23.06 (3.62) |
| Smoking (%) | No | 1,734 (73.98) | 1,294 (77.39) | 360 (65.57) | 80 (65.04) |
|  | Yes | 610 (26.02) | 378 (22.61) | 189 (34.43) | 43 (34.96) |
| Alcohol drinking (%) | No | 1,832 (78.16) | 1,364 (81.58) | 390 (71.04) | 78 (63.41) |
|  | Yes | 512 (21.84) | 308 (18.42) | 159 (28.96) | 45 (36.59) |
| * Advanced neoplasm (CRC cases and advanced adenoma); SD, Standard Deviation | | | | | |

| **Table S4. Basic characteristics of the PLCO cross-sectional screening set** | | | | | | |
| --- | --- | --- | --- | --- | --- | --- |
| Variables | |  | All participants | Normal | Non-advanced adenoma | Advanced neoplasm |
| N | |  | 24,322 | 17,713 | 3,946 | 2,663 |
| Sex (%) | | Male | 13,522 (55.60) | 9,314 (52.58) | 2,512 (63.66) | 1,696 (63.69) |
|  | | Female | 10,800 (44.40) | 8,399 (47.42) | 1,434 (36.34) | 967 (36.31) |
| Age (year), mean ± SD | | | 62.07 (5.21) | 61.90 (5.16) | 62.20 (5.25) | 63.04 (5.32) |
| BMI (kg/m3), mean ± SD | | | 27.41 (4.73) | 27.25 (4.73) | 27.89 (4.70) | 27.81 (4.69) |
| Smoking (%) | | No | 12,062 (49.78) | 9,430 (53.37) | 1,562 (39.87) | 1,070 (40.48) |
|  | | Yes | 12,169 (50.22) | 8,240 (46.6) | 2,356 (60.13) | 1,573 (59.52) |
|  | | missing | 91(0.37) | 43(0.24) | 28(0.70) | 20(0.75) |
| Alcohol drinking (%) | | No | 8,382 (34.46) | 6,207 (35.04) | 1,260 (31.93) | 915 (34.36) |
|  | | Yes | 15,940 (65.54) | 11506 (64.96) | 2,686 (68.07) | 1,748 (65.64) |
| * Advanced neoplasm (CRC cases and advanced adenoma); SD, Standard Deviation | | | | | |  |

| **Table S5. Basic characteristics of the PLCO incident adenoma cohort** | | | | | |
| --- | --- | --- | --- | --- | --- |
| Variables |  | All participants | Normal | Non-advanced adenoma | Advanced neoplasm |
| N |  | 15,992 | 14,922 | 701 | 369 |
| Sex (%) | Female | 8,550 (53.46) | 7,881 (52.81) | 437 (62.34) | 232 (62.87) |
|  | Male | 7,442 (46.54) | 7,041 (47.19) | 264 (37.66) | 137 (37.13) |
| Age(year), mean ± SD^a^ | | 61.85 (5.15) | 61.84 (5.13) | 61.33 (5.13) | 63.16 (5.63) |
| BMI (kg/m3), mean ± SD | | 27.41 (4.73) | 27.25 (4.73) | 27.89 (4.70) | 27.81 (4.69) |
| Smoking (%) | No | 8,650 (54.09) | 8,157 (54.66) | 319 (45.51) | 174 (47.15) |
|  | Yes | 7,342 (45.91) | 6,765 (45.34) | 382 (54.49) | 195 (52.85) |
| Alcohol drinking (%) | No | 5,457 (34.12) | 5,138 (34.43) | 203 (28.96) | 116 (31.44) |
|  | Yes | 10,535 (65.88) | 9,784 (65.57) | 498 (71.04) | 253 (68.56) |
| * Advanced neoplasm (CRC cases and advanced adenoma); SD, Standard Deviation  ^a^ Age at baseline. | | | | | |

| **Table S6. Basic characteristics of the UK Biobank cohort** | | | | |
| --- | --- | --- | --- | --- |
| Variables |  | All participants | Normal | Advanced neoplasm |
| N |  | 334,021 | 331,041 | 2,980 |
| Sex (%) | Female | 185,815 (55.63) | 184,559 (55.75) | 1,256 (42.15) |
|  | Male | 148,206 (44.37) | 146,482 (44.25) | 1,724 (57.85) |
| Age(year), mean ± SD^a^ | | 55.70 (8.08) | 55.65 (8.08) | 60.79 (6.41) |
| BMI (kg/m3), mean ± SD | | 27.28 (4.74) | 27.27 (4.74) | 27.89 (4.63) |
| Smoking (%) | No | 186,485 (55.83) | 185,103 (55.92) | 1,382 (46.38) |
|  | Yes | 147,536 (44.17) | 145,938 (44.08) | 1,598 (53.62) |
| Alcohol drinking (%) | No | 21,231 (6.36) | 21,032 (6.35) | 199 (6.68) |
|  | Yes | 312,790 (93.64) | 310,009 (93.65) | 2,781 (93.32) |
| * Advanced neoplasm (CRC cases and advanced adenoma); SD, Standard Deviation  ^a^ Age at baseline. | | | | |

| **Table S7. Imputation quality control information** | | | | | |
| --- | --- | --- | --- | --- | --- |
| Case-control/Cohort population | Platform | Pre-imputation QC | | | |
|  |  | MAF | HWE *P*-value | Call rate | Other exclusions |
| ZJCRC case-control set | Infinium Asian Screening Array | >0.01% | >10^-6^ | >95% | variants located on X/Y Chromosome |
| ZJCRC cross-sectional screening set |  |  |  |  |  |
| PLCO cross-sectional screening set | Illumina Global Screening Array | >0.01% | >10^-6^ | >95% | variants located on X/Y Chromosome |
| PLCO incident adenoma cohort | Illumina Oncoarray | >0.01% | >10^-6^ | >95% | variants located on X/Y Chromosome |
|  | Illumina Omni25 | >0.01% | >10^-6^ | >95% | variants located on X/Y Chromosome |

| Case-control/Cohort population | Platform | Imputation | |
| --- | --- | --- | --- |
|  |  | Tool | Panel |
| ZJCRC case-control set | Infinium Asian Screening Array | Michigan Imputation Server | 1000G Phase 3 v5(GRCh37/hg19) |
| ZJCRC cross-sectional screening set |  |  |  |
| PLCO cross-sectional screening set | Illumina Global Screening Array | TOPMed Imputation Server | TOPMed r2 |
| PLCO incident adenoma cohort | Illumina Oncoarray | TOPMed Imputation Server | TOPMed r2 |
|  | Illumina Omni25 | TOPMed Imputation Server | TOPMed r2 |

| Case-control/Cohort population | Platform | Post-imputation QC | | | |
| --- | --- | --- | --- | --- | --- |
|  |  | Quality | MAF | HWE *P*-value | Call rate |
| ZJCRC case-control set | Infinium Asian Screening Array | R^2^>0.4 | >0.1% | >10^-6^ | >95% |
| ZJCRC cross-sectional screening set |  |  |  |  |  |
| PLCO cross-sectional screening set | Illumina Global Screening Array | R^2^>0.4 | >0.1% | >10^-6^ | >95% |
| PLCO incident adenoma cohort | Illumina Oncoarray | R^2^>0.4 | >0.1% | >10^-6^ | >95% |
|  | Illumina Omni25 | R^2^>0.4 | >0.1% | >10^-6^ | >95% |
| * QC, quality control; MAF, Minor Allele Frequency; HWE, Hardy. Weinberg Equilibrium | | | | | |

| **Table S8. The discriminatory accuracy of SNPs filtered by different *P* thresholds and LD using 400 times 5-fold cross-validation** | | | | | |
| --- | --- | --- | --- | --- | --- |
| LD (r^2^) | *P* Threshold | AUC | Accuracy (SD) | Precision (SD) | Recall (SD) |
| 0.1 | 5×10^–2^ | 0.529 | 0.515 (0.019) | 0.518 (0.031) | 0.494 (0.075) |
|  | 5×10^–3^ | 0.550 | 0.532 (0.019) | 0.533 (0.030) | 0.516 (0.052) |
|  | 5×10^–4^ | 0.583 | 0.551 (0.020) | 0.553 (0.031) | 0.538 (0.042) |
|  | 5×10^–5^ | 0.594 | 0.568 (0.019) | 0.573 (0.031) | 0.538 (0.041) |
|  | 5×10^–6^ | 0.609 | 0.580 (0.019) | 0.580 (0.031) | 0.579 (0.034) |
|  | 5×10^–7^ | 0.603 | 0.572 (0.019) | 0.572 (0.030) | 0.573 (0.036) |
|  | **5×10^–8^** | **0.611** | **0.584 (0.019)** | **0.583 (0.029)** | **0.588 (0.036)** |
| 0.01 | 5×10^–2^ | 0.534 | 0.523 (0.020) | 0.527 (0.032) | 0.496 (0.073) |
|  | 5×10^–3^ | 0.546 | 0.528 (0.019) | 0.53 (0.0310) | 0.514 (0.053) |
|  | 5×10^–4^ | 0.574 | 0.550 (0.020) | 0.552 (0.033) | 0.538 (0.043) |
|  | 5×10^–5^ | 0.586 | 0.560 (0.020) | 0.566 (0.030) | 0.511 (0.042) |
|  | 5×10^–6^ | 0.609 | 0.576 (0.019) | 0.576 (0.030) | 0.575 (0.035) |
|  | 5×10^–7^ | 0.603 | 0.566 (0.020) | 0.565 (0.033) | 0.567 (0.034) |
|  | 5×10^–8^ | 0.606 | 0.575 (0.019) | 0.574 (0.031) | 0.579 (0.033) |
| 0.2 | 5×10^–2^ | 0.535 | 0.524 (0.020) | 0.527 (0.033) | 0.505 (0.065) |
|  | 5×10^–3^ | 0.559 | 0.537 (0.019) | 0.538 (0.030) | 0.522 (0.048) |
|  | 5×10^–4^ | 0.587 | 0.557 (0.020) | 0.559 (0.030) | 0.54 (0.042) |
|  | 5×10^–5^ | 0.596 | 0.571 (0.019) | 0.575 (0.033) | 0.543 (0.039) |
|  | 5×10^–6^ | 0.608 | 0.580 (0.019) | 0.579 (0.030) | 0.584 (0.034) |
|  | 5×10^–7^ | 0.606 | 0.577 (0.020) | 0.576 (0.031) | 0.580 (0.036) |
|  | 5×10^–8^ | 0.608 | 0.579 (0.019) | 0.579 (0.029) | 0.576 (0.037) |
| *LD, linkage disequilibrium; AUC, Area Under Curve; SD, Standard Deviation | | | | | |

| Table S9. Summary of 148 CRC reported GWAS SNPs in European and East Asian population | | | | | | | |
| --- | --- | --- | --- | --- | --- | --- | --- |
| SNP | Chromosome | Position (GRCh37) | Risk allele | Reference allele | P | OR | Ethnicity backgroud |
| rs72647484 | 1 | 22,587,728 | T | C | 1.40E-03 | 1.08 | European population |
| rs4360494 | 1 | 38,455,891 | G | C | 3.80E-09 | 1.05 | European population |
| rs12144319 | 1 | 55,246,035 | C | T | 3.30E-11 | 1.07 | European population |
| rs7542665 | 1 | 62673037 | C | T | 3.51E-08 | 1.08 | Asian population |
| rs6678517 | 1 | 183,002,639 | A | G | 5.30E-14 | 1.1 | European population |
| rs10911251 | 1 | 183081194 | A | C | 1.03E-04 | 1.05 | Asian population |
| rs17011141 | 1 | 222,112,634 | G | A | 3.20E-08 | 1.09 | European population |
| rs201395236 | 1 | 245181421 | T | C | 4.63E-08 | 1.75 | Asian population |
| rs7606562 | 2 | 48686695 | T | A | 1.21E-08 | 1.1 | Asian population |
| rs448513 | 2 | 159,964,552 | C | T | 4.40E-08 | 1.05 | European population |
| rs11884596 | 2 | 199,612,407 | C | T | 3.60E-09 | 1.06 | European population |
| rs983402 | 2 | 199,781,586 | T | C | 7.70E-12 | 1.07 | European population |
| rs992157 | 2 | 219154781 | A | G | 6.26E-03 | 1.04 | Asian population |
| rs3731861 | 2 | 219,191,256 | T | C | 1.10E-06 | 1.07 | European population |
| rs35470271 | 3 | 40,915,239 | G | A | 9.50E-05 | 1.07 | European population |
| rs35360328 | 3 | 40924962 | A | T | 1.00E-02 | 1.09 | Asian population |
| rs6781752 | 3 | 66,365,163 | A | G | 1.90E-03 | 1.05 | European population |
| rs72942485 | 3 | 112,999,560 | G | A | 2.10E-08 | 1.19 | European population |
| rs10049390 | 3 | 133,701,119 | A | G | 3.80E-09 | 1.06 | European population |
| rs113569514 | 3 | 133748789 | T | C | 2.45E-12 | 1.1 | Asian population |
| rs10936599 | 3 | 169492101 | C | T | 2.41E-06 | 1.06 | Asian population |
| rs9876206 | 3 | 169,517,436 | C | T | 9.90E-06 | 1.07 | European population |
| rs13149359 | 4 | 94,938,618 | A | C | 2.30E-04 | 1.05 | European population |
| rs1391441 | 4 | 106,128,760 | A | G | 1.60E-08 | 1.05 | European population |
| rs11727676 | 4 | 145,659,064 | C | T | 2.90E-08 | 1.09 | European population |
| rs78368589 | 5 | 1,240,204 | T | C | 4.10E-09 | 1.12 | European population |
| rs2735940 | 5 | 1,296,486 | G | A | 3.60E-11 | 1.09 | European/Asian population |
| rs7708610 | 5 | 40,102,443 | A | G | 3.80E-09 | 1.06 | European population |
| rs12514517 | 5 | 40,280,076 | A | G | 1.40E-12 | 1.1 | European population |
| rs58791712 | 5 | 40281798 | GT | G | 2.00E-02 | 1.12 | Asian population |
| rs145364999 | 5 | 98,206,082 | T | A | 6.30E-09 | 1.74 | European population |
| rs755229494 | 5 | 112,097,351 | G | A | 2.30E-02 | 2.01 | European population |
| rs12659017 | 5 | 125988175 | G | A | 4.45E-08 | 1.09 | Asian population |
| rs4976270 | 5 | 134,467,220 | C | T | 2.40E-07 | 1.07 | European population |
| rs647161 | 5 | 134499092 | A | C | 3.10E-15 | 1.12 | Asian population |
| rs1476570 | 6 | 29809860 | A | G | 6.71E-09 | 1.12 | Asian population |
| rs2516420 | 6 | 31,449,620 | C | T | 2.00E-10 | 1.12 | European population |
| rs3830041 | 6 | 32191339 | T | C | 1.65E-08 | 1.16 | Asian population |
| rs9271695 | 6 | 32,593,080 | G | A | 1.10E-13 | 1.09 | European population |
| rs16878812 | 6 | 35,569,562 | A | G | 1.20E-03 | 1.07 | European population |
| rs1321311 | 6 | 36622900 | A | C | 1.35E-06 | 1.09 | Asian population |
| rs9470361 | 6 | 36,623,379 | A | G | 1.30E-05 | 1.07 | European population |
| rs4711689 | 6 | 41692812 | A | G | 6.17E-06 | 1.09 | Asian population |
| rs62396735 | 6 | 41,702,582 | C | T | 5.80E-03 | 1.04 | European population |
| rs62404966 | 6 | 55,712,124 | C | T | 3.80E-05 | 1.06 | European population |
| rs62404968 | 6 | 55714314 | C | T | 3.00E-02 | 1.08 | Asian population |
| rs12672022 | 7 | 45,136,423 | T | C | 2.80E -8 | 1.07 | European population |
| rs2450115 | 8 | 117624093 | T | C | 2.39E-14 | 1.11 | Asian population |
| rs16892766 | 8 | 117,630,683 | C | A | 1.20E-16 | 1.21 | European population |
| rs6469654 | 8 | 117,632,965 | G | C | 4.00E-08 | 1.07 | European population |
| rs6469656 | 8 | 117647788 | A | G | 2.06E-10 | 1.09 | Asian population |
| rs117079142 | 8 | 117,790,914 | A | C | 8.30E-07 | 1.12 | European population |
| rs6983267 | 8 | 128,413,305 | G | T | 9.50E-36 | 1.17 | European/Asian population |
| rs4313119 | 8 | 128,571,855 | G | T | 2.10E-09 | 1.06 | European population |
| rs1537372 | 9 | 22,103,183 | G | T | 1.40E-08 | 1.05 | European population |
| rs34405347 | 9 | 101,679,752 | T | G | 3.10E-08 | 1.09 | European population |
| rs10980628 | 9 | 113,671,403 | C | T | 2.80E-09 | 1.07 | European population |
| rs10795668 | 10 | 8701219 | G | A | 5.20E-24 | 1.14 | Asian population |
| rs11255841 | 10 | 8,739,580 | T | A | 2.40E-12 | 1.1 | European population |
| rs10821907 | 10 | 52,648,454 | C | T | 9.90E-05 | 1.07 | European population |
| rs704017 | 10 | 80,819,132 | G | A | 1.50E-09 | 1.08 | European/Asian population |
| rs6584283 | 10 | 101290301 | C | T | 1.21E-08 | 1.09 | Asian population |
| rs1035209 | 10 | 101345366 | T | C | 1.12E-05 | 1.08 | Asian population |
| rs11190164 | 10 | 101,351,704 | G | A | 1.10E-06 | 1.07 | European population |
| rs4919687 | 10 | 104595248 | G | A | 2.94E-04 | 1.07 | Asian population |
| rs12241008 | 10 | 114280702 | C | T | 1.77E-11 | 1.11 | Asian population |
| rs12246635 | 10 | 114,288,619 | C | T | 1.40E-09 | 1.14 | European population |
| rs11196170 | 10 | 114,722,621 | A | G | 1.50E-03 | 1.05 | European population |
| rs11196172 | 10 | 114726843 | A | G | 3.19E-15 | 1.12 | Asian population |
| rs174533 | 11 | 61,549,025 | G | A | 1.20E-03 | 1.05 | European population |
| rs174537 | 11 | 61552680 | G | T | 7.52E-12 | 1.1 | Asian population |
| rs7121958 | 11 | 74,280,012 | G | T | 8.80E-10 | 1.08 | European population |
| rs3824999 | 11 | 74345550 | G | T | 3.16E-06 | 1.07 | Asian population |
| rs61389091 | 11 | 74,427,921 | C | T | 3.70E-16 | 1.21 | European population |
| rs2186607 | 11 | 101,656,397 | T | A | 1.50E-09 | 1.05 | European population |
| rs3087967 | 11 | 111,156,836 | T | C | 1.20E-18 | 1.13 | European population |
| rs3802842 | 11 | 111171709 | C | A | 5.00E-07 | 1.07 | Asian population |
| rs10774214 | 12 | 4368352 | T | C | 2.60E-09 | 1.09 | Asian population |
| rs35808169 | 12 | 4,368,607 | C | T | 1.00E-03 | 1.06 | European population |
| rs3217810 | 12 | 4,388,271 | T | C | 4.00E-08 | 1.12 | European population |
| rs3217874 | 12 | 4,400,808 | T | C | 2.40E-09 | 1.06 | European population |
| rs10849432 | 12 | 6385727 | T | C | 1.95E-05 | 1.08 | Asian population |
| rs2250430 | 12 | 6,421,174 | T | A | 7.50E-05 | 1.06 | European population |
| rs11064437 | 12 | 6982162 | C | T | 3.47E-03 | 1.04 | Asian population |
| rs2238126 | 12 | 12009741 | G | A | 3.00E-02 | 1.03 | Asian population |
| rs2710310 | 12 | 12,035,649 | C | T | 2.30E-02 | 1.03 | European population |
| rs77969132 | 12 | 31594813 | T | C | 4.85E-08 | 1.44 | Asian population |
| rs2730985 | 12 | 43130624 | G | A | 1.23E-08 | 1.08 | Asian population |
| rs11610543 | 12 | 43,134,191 | G | A | 1.30E-09 | 1.05 | European population |
| rs11169552 | 12 | 51155663 | C | T | 8.30E-03 | 1.04 | Asian population |
| rs12372718 | 12 | 51,171,090 | G | A | 2.90E-12 | 1.1 | European population |
| rs4759277 | 12 | 57,533,690 | A | C | 9.40E-09 | 1.05 | European population |
| rs597808 | 12 | 111,973,358 | G | A | 5.80E-07 | 1.07 | European population |
| rs72013726 | 12 | 115890836 | C | CACAA | 1.00E-02 | 1.05 | Asian population |
| rs7300312 | 12 | 115,890,922 | C | T | 9.20E-07 | 1.07 | European population |
| rs377429877 | 13 | 34,092,164 | C | T | 1.80E-02 | 1.03 | European population |
| rs7333607 | 13 | 37,462,010 | G | A | 6.30E-13 | 1.08 | European population |
| rs78341008 | 13 | 73,791,554 | C | T | 3.20E-10 | 1.12 | European population |
| rs1886450 | 13 | 73986628 | G | A | 6.28E-12 | 1.09 | Asian population |
| rs8000189 | 13 | 111,075,881 | T | C | 1.80E-09 | 1.06 | European population |
| rs4444235 | 14 | 54410919 | C | T | 7.85E-04 | 1.04 | Asian population |
| rs35107139 | 14 | 54,419,106 | C | A | 9.90E-12 | 1.09 | European population |
| rs4901473 | 14 | 54,445,157 | G | A | 2.60E-07 | 1.05 | European population |
| rs17094983 | 14 | 59,189,361 | G | A | 4.60E-11 | 1.09 | European population |
| rs12708491 | 15 | 32,992,836 | G | A | 9.00E-07 | 1.05 | European population |
| rs16969681 | 15 | 32993111 | T | C | 1.31E-08 | 1.08 | Asian population |
| rs4779584 | 15 | 32994756 | T | C | 2.00E-02 | 1.04 | Asian population |
| rs2293581 | 15 | 33,010,736 | A | G | 1.10E-29 | 1.13 | European population |
| rs17816465 | 15 | 33,156,386 | A | G | 1.40E-10 | 1.07 | European population |
| rs56324967 | 15 | 67,402,824 | C | T | 1.10E-13 | 1.07 | European population |
| rs9924886 | 16 | 68,743,939 | A | C | 2.40E-04 | 1.05 | European population |
| rs9929218 | 16 | 68820946 | G | A | 9.22E-03 | 1.05 | Asian population |
| rs4341754 | 16 | 80039621 | G | C | 1.73E-09 | 1.09 | Asian population |
| rs9930005 | 16 | 80,043,258 | C | A | 2.10E-08 | 1.05 | European population |
| rs847208 | 16 | 86254051 | A | C | 2.62E-07 | 1.08 | Asian population |
| rs12149163 | 16 | 86,339,315 | T | C | 5.50E-04 | 1.04 | European population |
| rs2696839 | 16 | 86340448 | G | C | 4.00E-02 | 1.03 | Asian population |
| rs62042090 | 16 | 86,703,949 | T | C | 2.40E-04 | 1.06 | European population |
| rs12603526 | 17 | 800593 | C | T | 6.40E-05 | 1.06 | Asian population |
| rs4968127 | 17 | 809,643 | G | A | 4.00E-07 | 1.07 | European population |
| rs1078643 | 17 | 10,707,241 | A | G | 6.60E-12 | 1.08 | European/Asian population |
| rs983318 | 17 | 70,413,253 | A | G | 5.60E-09 | 1.06 | European population |
| rs75954926 | 17 | 81,061,048 | G | A | 3.00E-18 | 1.09 | European population |
| rs7229639 | 18 | 46450976 | A | G | 2.49E-27 | 1.21 | Asian population |
| rs11874392 | 18 | 46,453,156 | A | T | 2.10E-41 | 1.19 | European population |
| rs4939827 | 18 | 46453463 | T | C | 5.44E-15 | 1.13 | Asian population |
| rs34797592 | 19 | 16,417,198 | T | C | 4.20E-10 | 1.09 | European population |
| rs28840750 | 19 | 33,519,927 | T | G | 2.60E-10 | 1.21 | European population |
| rs10411210 | 19 | 33532300 | C | T | 1.22E-07 | 1.1 | Asian population |
| rs1800469 | 19 | 41860296 | G | A | 1.61E-05 | 1.06 | Asian population |
| rs1963413 | 19 | 41,871,573 | A | G | 1.50E-04 | 1.05 | European population |
| rs73068325 | 19 | 59,079,096 | T | C | 4.20E-08 | 1.07 | European population |
| rs189583 | 20 | 6,376,457 | G | C | 3.10E-11 | 1.09 | European population |
| rs961253 | 20 | 6404281 | A | C | 6.93E-04 | 1.08 | Asian population |
| rs994308 | 20 | 6,603,622 | C | T | 8.60E-12 | 1.06 | European population |
| rs4813802 | 20 | 6,699,595 | G | T | 4.60E-07 | 1.07 | European/Asian population |
| rs28488 | 20 | 6,762,221 | T | C | 2.60E-14 | 1.07 | European population |
| rs11087784 | 20 | 7,740,976 | G | A | 2.20E-07 | 1.1 | European population |
| rs2423279 | 20 | 7812350 | C | T | 2.87E-10 | 1.1 | Asian population |
| rs6058093 | 20 | 33,213,196 | C | A | 1.70E-02 | 1.03 | European population |
| rs6065668 | 20 | 42532821 | T | C | 3.65E-07 | 1.07 | Asian population |
| rs6031311 | 20 | 42,666,475 | T | C | 6.80E-09 | 1.06 | European population |
| rs6066825 | 20 | 47,340,117 | A | G | 1.70E-08 | 1.08 | European/Asian population |
| rs6063514 | 20 | 49,055,318 | C | T | 7.30E-07 | 1.07 | European population |
| rs1810502 | 20 | 49057488 | C | T | 1.38E-06 | 1.07 | Asian population |
| rs13831 | 20 | 57475191 | G | A | 2.06E-08 | 1.08 | Asian population |
| rs1741640 | 20 | 60,932,414 | C | T | 3.90E-09 | 1.1 | European population |
| rs2738783 | 20 | 62,308,612 | T | G | 5.30E-08 | 1.06 | European population |

| **Table S10. Summary of 183 selected SNPs of European and East Asian CRC GWAS meta** | | | | | | |
| --- | --- | --- | --- | --- | --- | --- |
| SNP | Chromosome | Position (GRCh37) | Risk allele | Reference allele | *P* | BETA |
| rs371061408 | 1 | 222147550 | T | C | 5.89E-28 | -0.1212 |
| rs17011146 | 1 | 222113648 | A | G | 1.00E-18 | -0.0948 |
| rs34099011 | 1 | 183085210 | A | G | 4.74E-16 | 0.0667 |
| rs12120034 | 1 | 222142975 | A | G | 5.87E-15 | 0.0823 |
| rs28605759 | 1 | 38399816 | A | G | 7.66E-13 | -0.0580 |
| rs12143541 | 1 | 55247852 | A | G | 7.30E-12 | -0.0844 |
| rs9441981 | 1 | 222011188 | T | C | 2.25E-11 | -0.0635 |
| rs6673317 | 1 | 222074742 | A | G | 4.12E-11 | 0.0633 |
| rs1379647056 | 1 | 222093616 | G | GTA | 8.28E-11 | -0.0725 |
| rs6658977 | 1 | 222049820 | T | G | 1.60E-10 | 0.0592 |
| rs2270003 | 1 | 55248702 | A | C | 3.80E-10 | 0.0561 |
| rs9729284 | 1 | 38373989 | T | C | 1.67E-09 | 0.0534 |
| rs6674188 | 1 | 55287633 | A | G | 6.29E-09 | 0.0921 |
| rs78452026 | 1 | 55277198 | A | G | 6.46E-09 | 0.0832 |
| rs6679981 | 1 | 22697860 | A | G | 6.67E-09 | -0.0612 |
| rs112191583 | 1 | 22554378 | T | C | 2.79E-08 | -0.1979 |
| rs72729468 | 1 | 183104746 | A | C | 2.79E-08 | -0.0578 |
| rs4233286 | 1 | 22688589 | T | C | 2.91E-08 | 0.0518 |
| rs7557709 | 2 | 219167529 | A | G | 4.81E-16 | 0.0679 |
| rs7572632 | 2 | 199829150 | T | C | 3.82E-11 | 0.0619 |
| rs1446646 | 2 | 200243869 | T | C | 3.74E-10 | -0.0606 |
| rs1917932 | 2 | 199752613 | A | G | 1.24E-09 | 0.0598 |
| rs7564883 | 2 | 199796797 | T | C | 3.40E-09 | 0.0542 |
| rs3192177 | 2 | 98354511 | A | G | 1.61E-08 | -0.0531 |
| rs7564773 | 2 | 98571084 | A | G | 1.65E-08 | 0.0530 |
| rs17572109 | 2 | 219093934 | A | G | 1.68E-08 | -0.0641 |
| rs1701530871 | 2 | 199612126 | G | GA | 3.04E-08 | 0.0467 |
| rs35783535 | 2 | 98623227 | A | G | 3.14E-08 | -0.0528 |
| rs17488834 | 2 | 98354139 | T | G | 3.79E-08 | -0.0497 |
| rs11707109 | 3 | 40914588 | A | G | 6.07E-14 | -0.0927 |
| rs72942485 | 3 | 112999560 | A | G | 7.17E-12 | -0.1623 |
| s6808387 | 3 | 53079732 | T | C | 3.78E-11 | 0.0588 |
| rs77572134 | 3 | 52895934 | T | C | 5.06E-10 | 0.0799 |
| rs73864033 | 3 | 133736982 | T | C | 1.19E-09 | -0.0743 |
| rs6768530 | 3 | 64611332 | T | C | 1.22E-09 | -0.0489 |
| rs7614538 | 3 | 133707001 | A | G | 2.33E-09 | -0.0600 |
| rs6599127 | 3 | 40957691 | T | C | 4.76E-09 | 0.0570 |
| rs13086367 | 3 | 112903888 | A | G | 1.50E-08 | 0.0464 |
| rs1736339795 | 4 | 175419866 | G | GT | 7.60E-10 | -0.0502 |
| rs10022844 | 4 | 151499440 | A | G | 3.64E-09 | -0.0470 |
| rs17365288 | 4 | 94904738 | A | G | 1.44E-08 | -0.0474 |
| rs4698928 | 4 | 105852553 | T | C | 3.17E-08 | 0.0455 |
| rs2735940 | 5 | 1296486 | A | G | 8.51E-29 | -0.0917 |
| rs1445011 | 5 | 40280202 | T | C | 1.28E-25 | -0.1023 |
| rs4976270 | 5 | 134467220 | T | C | 1.27E-22 | -0.0802 |
| rs4583925 | 5 | 1249047 | T | C | 1.93E-16 | 0.1518 |
| rs7728240 | 5 | 134431949 | T | C | 5.97E-10 | 0.0526 |
| rs6911915 | 6 | 117809031 | T | C | 1.75E-13 | -0.0617 |
| rs62404966 | 6 | 55712124 | T | C | 5.97E-13 | -0.0741 |
| rs16878812 | 6 | 35569562 | A | G | 8.73E-11 | 0.0964 |
| rs9462210 | 6 | 36628953 | A | G | 2.21E-09 | 0.0581 |
| rs1447129 | 6 | 55703942 | T | C | 8.79E-09 | 0.0475 |
| rs2516452 | 6 | 31427095 | A | G | 9.57E-09 | 0.0806 |
| rs9264796 | 6 | 31270311 | A | G | 1.05E-08 | 0.1085 |
| rs9264943 | 6 | 31274521 | A | G | 4.12E-08 | 0.0777 |
| rs114363880 | 7 | 45119390 | A | G | 4.66E-11 | 0.0875 |
| rs4727436 | 7 | 99486165 | T | C | 2.20E-10 | -0.0541 |
| rs3801081 | 7 | 47511161 | A | G | 8.05E-10 | -0.0547 |
| rs12666319 | 7 | 46884575 | A | G | 3.54E-08 | -0.0502 |
| rs6983267 | 8 | 128413305 | T | G | 3.13E-77 | -0.1502 |
| rs2437844 | 8 | 117622571 | A | G | 1.86E-28 | 0.1326 |
| rs28774977 | 8 | 128430807 | A | C | 2.22E-20 | 0.1024 |
| rs75128197 | 8 | 117734589 | A | C | 7.52E-19 | 0.1823 |
| rs72712390 | 8 | 128429652 | A | G | 1.46E-15 | -0.0967 |
| rs1562871 | 8 | 128401772 | T | C | 1.98E-15 | -0.0767 |
| rs2015069 | 8 | 117639532 | T | C | 4.58E-15 | 0.0834 |
| rs79101886 | 8 | 128413783 | T | G | 2.50E-11 | 0.1072 |
| rs4493936 | 8 | 117760945 | T | C | 2.72E-11 | 0.0765 |
| rs181112261 | 8 | 117765022 | A | G | 3.70E-10 | 0.0705 |
| rs200141259 | 8 | 128429456 | G | GAAAGA | 1.54E-09 | 0.0740 |
| rs944796 | 9 | 22115286 | T | C | 9.34E-13 | 0.0571 |
| rs61237993 | 9 | 34130435 | A | G | 4.15E-11 | 0.0711 |
| rs56270678 | 9 | 33841827 | A | C | 3.83E-10 | 0.0585 |
| rs6475604 | 9 | 22052734 | T | C | 2.86E-08 | 0.0475 |
| rs11255815 | 10 | 8714465 | T | C | 5.33E-31 | 0.1141 |
| rs704017 | 10 | 80819132 | A | G | 2.67E-21 | -0.0787 |
| rs2193352 | 10 | 101346609 | A | G | 1.42E-19 | -0.0929 |
| rs76880073 | 10 | 114276661 | A | G | 1.81E-15 | -0.1052 |
| rs11196173 | 10 | 114727067 | T | C | 9.93E-13 | 0.0772 |
| rs11255866 | 10 | 8757444 | T | C | 2.16E-12 | -0.0655 |
| 10_52646867_G_GT | 10 | 52646867 | G | GT | 1.08E-10 | 0.0813 |
| rs17500846 | 10 | 52642384 | A | G | 1.30E-10 | -0.0596 |
| rs72818736 | 10 | 91399564 | T | G | 2.87E-10 | 0.0686 |
| rs140356782 | 10 | 91505293 | T | C | 2.75E-09 | -0.0635 |
| rs6480917 | 10 | 80810343 | T | C | 6.00E-09 | -0.0635 |
| rs2111324 | 10 | 101396564 | A | G | 1.30E-08 | 0.0464 |
| rs3087967 | 11 | 111156836 | T | C | 1.34E-38 | 0.1141 |
| rs11213825 | 11 | 111180390 | A | C | 9.61E-25 | 0.1019 |
| rs11604752 | 11 | 74277144 | A | G | 5.88E-19 | 0.0713 |
| rs4944940 | 11 | 74415252 | A | G | 2.04E-15 | -0.1867 |
| rs61389091 | 11 | 74427921 | T | C | 3.40E-15 | -0.1864 |
| rs174576 | 11 | 61603510 | A | C | 3.75E-14 | -0.0628 |
| rs1815949 | 11 | 111232259 | T | C | 7.89E-12 | 0.0608 |
| rs2003311 | 11 | 111204671 | A | G | 1.26E-11 | -0.0570 |
| rs2002315 | 11 | 69941836 | T | C | 7.09E-10 | -0.0512 |
| rs11604951 | 11 | 74335121 | T | C | 5.40E-09 | 0.0704 |
| rs1949941870 | 11 | 100250452 | T | TA | 2.46E-08 | 0.2957 |
| rs1046872 | 11 | 111179390 | A | G | 3.19E-08 | 0.0461 |
| rs149288432 | 11 | 100414002 | A | G | 4.72E-08 | -0.3037 |
| rs34863430 | 12 | 51201758 | A | G | 1.23E-18 | -0.0731 |
| rs12818766 | 12 | 4376091 | A | G | 9.15E-17 | 0.0998 |
| rs11169484 | 12 | 50934931 | A | G | 5.60E-15 | 0.0673 |
| rs12822984 | 12 | 115888504 | A | G | 6.22E-15 | 0.0642 |
| rs671 | 12 | 112241766 | A | G | 1.74E-13 | -0.1468 |
| rs3217810 | 12 | 4388271 | T | C | 2.47E-13 | 0.1072 |
| rs1961622 | 12 | 51247003 | A | G | 5.76E-13 | -0.0621 |
| rs79105258 | 12 | 111718231 | A | C | 9.75E-13 | -0.1467 |
| rs3217874 | 12 | 4400808 | T | C | 1.33E-12 | 0.0583 |
| rs7302363 | 12 | 50761067 | T | C | 2.23E-12 | 0.0606 |
| rs3217870 | 12 | 4400111 | T | C | 2.61E-12 | -0.0591 |
| rs3217840 | 12 | 4394877 | T | C | 6.82E-12 | 0.0622 |
| rs11067228 | 12 | 115094260 | A | G | 4.00E-11 | -0.0540 |
| rs11108174 | 12 | 96050752 | T | C | 1.13E-10 | 0.0546 |
| rs7955205 | 12 | 6425927 | T | C | 2.11E-10 | 0.0534 |
| rs4767318 | 12 | 115874054 | A | G | 3.70E-10 | 0.0548 |
| rs2870841 | 12 | 71476684 | T | G | 9.98E-10 | 0.0497 |
| rs17124432 | 12 | 50510777 | T | C | 3.26E-09 | 0.0521 |
| rs61928263 | 12 | 50588659 | T | C | 5.48E-09 | -0.0630 |
| rs11610543 | 12 | 43134191 | A | G | 5.54E-09 | -0.0467 |
| rs199879110 | 12 | 111377776 | CT | C | 6.53E-09 | -0.1232 |
| rs11066453 | 12 | 113365621 | A | G | 3.47E-08 | 0.1291 |
| rs200713222 | 12 | 120434410 | T | TCAGA | 4.16E-08 | -0.056 |
| rs4119480 | 13 | 37472079 | T | C | 5.36E-12 | -0.0725 |
| rs7320268 | 13 | 34079866 | A | G | 1.69E-10 | 0.0545 |
| rs45597035 | 13 | 73649152 | A | G | 4.47E-10 | 0.0537 |
| rs61966314 | 13 | 73771195 | A | G | 4.78E-08 | 0.0908 |
| rs35107139 | 14 | 54419106 | A | C | 2.07E-23 | -0.0857 |
| rs11157780 | 14 | 51371130 | T | C | 8.12E-16 | 0.0756 |
| rs17563 | 14 | 54417522 | A | G | 5.65E-12 | 0.0573 |
| rs2048123439 | 14 | 59204520 | A | AGTT | 5.42E-11 | -0.1122 |
| rs2293581 | 15 | 33010736 | A | G | 9.64E-30 | 0.1058 |
| rs16970016 | 15 | 32995298 | A | C | 1.14E-29 | 0.1127 |
| rs10318 | 15 | 33025979 | T | C | 3.16E-20 | 0.0877 |
| rs7494781 | 15 | 32988138 | T | C | 1.21E-14 | 0.0875 |
| rs1554868 | 15 | 32999483 | A | G | 5.86E-13 | -0.0647 |
| rs57284764 | 15 | 33057566 | A | C | 1.92E-12 | 0.0817 |
| rs3809570 | 15 | 67000117 | A | C | 3.55E-10 | -0.0631 |
| rs1258756 | 15 | 33051810 | A | G | 1.00E-09 | 0.0541 |
| rs56324967 | 15 | 67402824 | T | C | 1.83E-09 | -0.0540 |
| rs12591992 | 15 | 32994056 | A | G | 2.13E-08 | -0.0478 |
| rs7194355 | 16 | 68770288 | A | C | 7.93E-12 | -0.0633 |
| rs12149163 | 16 | 86339315 | T | C | 4.04E-11 | 0.0544 |
| rs551355324 | 16 | 86269402 | T | C | 6.00E-09 | -0.0758 |
| rs28448276 | 16 | 86161667 | A | C | 6.44E-09 | 0.0528 |
| rs4505341 | 16 | 80034203 | A | C | 1.04E-08 | -0.0471 |
| rs12598725 | 16 | 86347342 | T | G | 4.67E-08 | -0.0617 |
| rs1078643 | 17 | 10707241 | A | G | 4.24E-13 | 0.0801 |
| rs78268366 | 17 | 811407 | A | C | 1.36E-11 | 0.0561 |
| rs983318 | 17 | 70413253 | A | G | 1.43E-09 | 0.0610 |
| rs77433872 | 17 | 81025744 | A | G | 1.43E-09 | -0.0707 |
| rs2337113 | 18 | 46452327 | A | G | 5.12E-78 | 0.1519 |
| rs6507877 | 18 | 46458950 | A | G | 6.52E-27 | 0.0871 |
| rs9946510 | 18 | 46458227 | A | C | 5.73E-20 | -0.0874 |
| rs4939824 | 18 | 46392391 | A | G | 2.37E-16 | 0.0781 |
| rs6507878 | 18 | 46463756 | T | C | 1.59E-15 | -0.0645 |
| 18_46420749_AC_A | 18 | 46420749 | A | AC | 1.19E-11 | 0.0744 |
| rs2635513 | 18 | 46504760 | T | G | 4.19E-11 | 0.0617 |
| rs8098041 | 18 | 46448875 | A | G | 2.84E-09 | 0.1078 |
| rs73039433 | 19 | 33524604 | A | G | 8.07E-19 | -0.1449 |
| rs6510329 | 19 | 33520048 | A | C | 8.06E-13 | 0.1733 |
| rs79379434 | 19 | 33561038 | A | G | 8.53E-09 | -0.1673 |
| rs10425324 | 19 | 33541302 | T | C | 8.84E-09 | -0.0543 |
| rs1741640 | 20 | 60932414 | T | C | 2.44E-35 | -0.1364 |
| rs4813802 | 20 | 6699595 | T | G | 4.84E-24 | -0.0884 |
| rs6117251 | 20 | 6406440 | A | G | 1.54E-22 | -0.0899 |
| rs6086196 | 20 | 7749957 | A | G | 6.07E-20 | -0.0841 |
| rs6067449 | 20 | 49056840 | T | C | 2.09E-18 | 0.0720 |
| rs6066825 | 20 | 47340117 | A | G | 2.97E-18 | 0.0737 |
| rs6020486 | 20 | 49026111 | A | G | 9.69E-14 | -0.0631 |
| rs13042028 | 20 | 6438282 | T | G | 4.04E-13 | 0.0611 |
| rs2236200 | 20 | 60986019 | A | C | 1.54E-12 | 0.0743 |
| 20_33079384_TAAAA_T | 20 | 33079384 | T | TAAAA | 7.33E-12 | -0.0613 |
| rs1321456 | 20 | 6599528 | A | G | 2.22E-11 | -0.0591 |
| rs11699816 | 20 | 48978609 | T | C | 2.23E-11 | -0.0553 |
| rs6017248 | 20 | 42658274 | A | C | 1.45E-10 | -0.0586 |
| rs17720145 | 20 | 6383710 | A | G | 2.96E-10 | -0.0593 |
| rs4811073 | 20 | 49099809 | T | C | 8.47E-10 | -0.0497 |
| rs6125934 | 20 | 48803937 | T | C | 3.07E-09 | -0.0494 |
| rs235770 | 20 | 6761765 | T | C | 4.29E-09 | -0.0497 |
| rs11696198 | 20 | 62298404 | A | G | 8.01E-09 | -0.0558 |
| rs235767 | 20 | 6755598 | T | G | 9.03E-09 | -0.0474 |
| rs6038521 | 20 | 6475234 | T | G | 1.05E-08 | 0.0561 |
| rs6091189 | 20 | 49256285 | T | C | 1.89E-08 | 0.0719 |

| **Table S11. The weights of environmental factors** | | | |
| --- | --- | --- | --- |
| Environmental factors | OR (95%CI) | *P* | Effect |
| Smoking | 1.68 (1.35-2.07) | 2.06×10^-6^ | 0.517 |
| (current/former *vs* no) |  |  |  |
| Alcohol drinking | 1.78 (1.46-2.18) | 1.48×10^-8^ | 0.577 |
| (current/former *vs* no) |  |  |  |
| Exercise | 1.15 (0.89-1.49) | 0.275 | 0.142 |
| (<= 4 *vs* >4 per week) |  |  |  |
| BMI | 1.16 (0.99-1.36) | 0.0730 | 0.147 |
| (>= 24 *vs* <24 kg/m^2^) |  |  |  |
| Dietary quality score | 1.20 (1.00-1.45) | 0.0558 | 0.183 |
| (<= 4 *vs* >4) |  |  |  |
| * All *P*-values were calculated by unconditional logistic regression model after adjusting for gender, age, family history. | | | |

| **Table S12. Summary of lifestyle factors of the ZJCRC case-control set** | | | | |
| --- | --- | --- | --- | --- |
| Environmental factors | Score | All participants | Normal | Advanced neoplasm |
| Smoking (%) | 0 | 2,083 (57.41) | 1,125 (62.02) | 958 (52.81) |
|  | 1 | 1,545 (42.59) | 689 (37.98) | 856 (47.19) |
| Alcohol drinking (%) | 0 | 2,470 (68.08) | 1,329 (73.26) | 1,141 (62.90) |
|  | 1 | 1,158 (31.92) | 485 (26.74) | 673 (37.10) |
| BMI (%) | 0 | 2,022 (55.73) | 1,035 (57.06) | 987 (54.41) |
|  | 1 | 1,606 (44.27) | 779 (42.94) | 827 (45.59) |
| Exercise (%) | 0 | 610 (16.81) | 297 (16.37) | 313 (17.25) |
|  | 1 | 3,018 (83.19) | 1,517 (83.63) | 1,501 (82.75) |
| Dietary quality score (%) | 0 | 1,838 (50.66) | 927 (51.10) | 911 (50.22) |
|  | 1 | 1,790 (49.34) | 887 (48.90) | 903 (49.78) |

| **Table S13. Summary of lifestyle factors of the ZJCRC cross-sectional screening set** | | | | | |
| --- | --- | --- | --- | --- | --- |
| Environmental factors | Score | All participants | Normal | Non-advanced adenoma | Advanced neoplasm |
| Smoking (%) | 0 | 1,646 (70.22) | 1,238 (74.04) | 332 (60.47) | 76 (61.79) |
|  | 1 | 698 (29.78) | 434 (25.96) | 217 (39.53) | 47 (38.21) |
| Alcohol drinking (%) | 0 | 1,774 (75.68) | 1,325 (79.25) | 375 (68.31) | 74 (60.16) |
|  | 1 | 570 (24.32) | 347 (20.75) | 174 (31.69) | 49 (39.84) |
| BMI (%) | 0 | 1,378 (58.79) | 987 (59.03) | 312 (56.83) | 79 (64.23) |
|  | 1 | 966 (41.21) | 685 (40.97) | 237 (43.17) | 44 (35.77) |
| Exercise (%) | 0 | 343 (14.63) | 244 (14.59) | 76 (13.84) | 23 (18.70) |
|  | 1 | 2,001 (85.37) | 1,428 (85.41) | 473 (86.16) | 100 (81.30) |
| Dietary quality score (%) | 0 | 1,340 (57.17) | 991 (59.27) | 280 (51.00) | 69 (56.10) |
|  | 1 | 1,004 (42.83) | 681 (40.73) | 269 (49.00) | 54 (43.90) |

| **Table S14. Summary of lifestyle factors of the PLCO cross-sectional screening set** | | | | | |
| --- | --- | --- | --- | --- | --- |
| Environmental factors | Score | All participants | Normal | Non-advanced adenoma | Advanced neoplasm |
| Smoking (%) | 0 | 12,092 (49.72) | 9,449 (53.35) | 1,568 (39.74) | 1,075 (40.37) |
|  | 1 | 12,230 (50.28) | 8,264 (46.65) | 2,378 (60.26) | 1,588 (59.63) |
| Alcohol drinking (%) | 0 | 8,382 (34.46) | 6,207 (35.04) | 1,260 (31.93) | 915 (34.36) |
|  | 1 | 15,940 (65.54) | 11,506 (64.96) | 2,686 (68.07) | 1,748 (65.64) |
| BMI (%) | 0 | 5,583 (22.95) | 4,305 (24.30) | 774 (19.61) | 504 (18.93) |
|  | 1 | 18,739 (77.05) | 13,408 (75.70) | 3,172 (80.39) | 2,159 (81.07) |
| Dietary quality score (%) | 0 | 12,161 (50.00) | 8,533 (48.17) | 2,194 (55.60) | 1,434 (53.85) |
|  | 1 | 12,161 (50.00) | 9,180 (51.83) | 1,752 (44.40) | 1,229 (46.15) |

| **Table S15. Summary of lifestyle factors of the PLCO incident adenoma cohort** | | | | | |
| --- | --- | --- | --- | --- | --- |
| Environmental factors | Score | All participants | Normal | Non-advanced adenoma | Advanced neoplasm |
| Smoking (%) | 0 | 8,650 (54.09) | 8,157 (54.66) | 319 (45.51) | 174 (47.15) |
|  | 1 | 7,342 (45.91) | 6,765 (45.34) | 382 (54.49) | 195 (52.85) |
| Alcohol drinking (%) | 0 | 5,457 (34.12) | 5,138 (34.43) | 203 (28.96) | 116 (31.44) |
|  | 1 | 10,535 (65.88) | 9,784 (65.57) | 498 (71.04) | 253 (68.56) |
| BMI (%) | 0 | 3,947 (24.68) | 3,721 (24.94) | 155 (22.11) | 71 (19.24) |
|  | 1 | 12,045 (75.32) | 11,201 (75.06) | 546 (77.89) | 298 (80.76) |
| Dietary quality score (%) | 0 | 7,701 (48.16) | 7,134 (47.81) | 385 (54.92) | 182 (49.32) |
|  | 1 | 8,291 (51.84) | 7,788 (52.19) | 316 (45.08) | 187 (50.68) |

| **Table S16. Summary of lifestyle factors of the PLCO incident adenoma cohort** | | | | |
| --- | --- | --- | --- | --- |
| Environmental factors | Score | All participants | Normal | Advanced neoplasm |
| Smoking (%) | 0 | 186,485 (55.83) | 185,103 (55.92) | 1,382 (46.38) |
|  | 1 | 147,536 (44.17) | 145,938 (44.08) | 1,598 (53.62) |
| Alcohol drinking (%) | 0 | 21,231 (6.36) | 21,032 (6.35) | 199 (6.68) |
|  | 1 | 312,790 (93.64) | 310,009 (93.65) | 2,781 (93.32) |
| BMI (%) | 0 | 82,973 (24.84) | 82,397 (24.89) | 576 (19.33) |
|  | 1 | 251,048 (75.16) | 248,644 (75.11) | 2,404 (80.67) |
| Exercise (%) | 0 | 315,496 (94.45) | 312,701 (94.46) | 2,795 (93.79) |
|  | 1 | 18,525 (5.55) | 18,340 (5.54) | 185 (6.21) |
| Dietary quality score (%) | 0 | 130,938 (39.20) | 129,880 (39.23) | 1,058 (35.50) |
|  | 1 | 203,083 (60.80) | 201,161 (60.77) | 1,922 (64.50) |

| **Table S17.** **Prediction accuracy of three approach of the contrasted trans-ancestry PRS (2% of the PRS distribution as classifier).** | | | | | |
| --- | --- | --- | --- | --- | --- |
| Population | PRS threshold: Top 2% versus other 98% | | | | |
|  | OR (95% CI), *P* | Sensitivity | Specificity | PPV | NPV |
| **Approach 1 (PRS_148_)** |  |  |  |  |  |
| ZJCRC case-control set |  |  |  |  |  |
| Advanced lesions vs Normal | 3.14 (1.83-5.37), *P*=2.99×10^-5^ | - | - | - | - |
| ZJCRC cross-sectional screening set |  |  |  |  |  |
| NAA vs Normal | 1.15 (0.58-2.27), *P*=0.694 | 0.02 | 0.98 | 0.29 | 0.75 |
| Advanced lesions vs NAA | 1.62 (0.43-6.16), *P*=0.478 | 0.02 | 0.98 | 0.21 | 0.82 |
| Advanced lesions vs NAA and Normal | 1.94 (0.67-5.64), *P*=0.225 | 0.03 | 0.98 | 0.09 | 0.95 |
| Advanced lesions vs Normal | 1.74 (0.58-5.21), *P*=0.323 | 0.03 | 0.98 | 0.11 | 0.93 |
| PLCO cross-sectional screening set |  |  |  |  |  |
| NAA vs Normal | 0.86 (0.65-1.13), *P*=0.276 | 0.02 | 0.98 | 0.15 | 0.82 |
| Advanced lesions vs NAA | 1.44 (1.02-2.04), *P*=0.0374 | 0.02 | 0.98 | 0.12 | 0.89 |
| Advanced lesions vs NAA and Normal | 1.12 (0.84-1.47), *P*=0.44 | 0.02 | 0.98 | 0.12 | 0.89 |
| Advanced lesions vs Normal | 1.08 (0.78-1.5), *P*=0.623 | 0.02 | 0.98 | 0.14 | 0.87 |
| **Approach 2 (PRS_183_)** |  |  |  |  |  |
| ZJCRC case-control set |  |  |  |  |  |
| Advanced lesions vs Normal | 2.91(1.71-4.93), *P*=7.37×10^-5^ | - | - | - | - |
| ZJCRC cross-sectional screening set |  |  |  |  |  |
| NAA vs Normal | 1.45 (0.74-2.82), *P*=0.276 | 0.02 | 0.98 | 0.3 | 0.75 |
| Advanced lesions vs NAA | 3.54 (1.16-10.76), *P*=0.0259 | 0.05 | 0.99 | 0.43 | 0.82 |
| Advanced lesions vs NAA and Normal | 4.40 (1.95-9.94), *P*= 3.57×10^-4^ | 0.07 | 0.98 | 0.17 | 0.95 |
| Advanced lesions vs Normal | 6.48 (2.82-14.91), *P*=1.1×10^-5^ | 0.07 | 0.98 | 0.25 | 0.94 |
| PLCO cross-sectional screening set |  |  |  |  |  |
| NAA vs Normal | 1.48 (1.19-1.85), *P*=4.80×10^-4^ | 0.03 | 0.98 | 0.25 | 0.82 |
| Advanced lesions vs NAA | 1.65 (1.1-2.45), *P*=0.0143 | 0.02 | 0.98 | 0.49 | 0.60 |
| Advanced lesions vs NAA and Normal | 2.1 (1.62-2.73), *P*=2.11×10^-8^ | 0.03 | 0.98 | 0.19 | 0.89 |
| Advanced lesions vs Normal | 2.33 (1.78-3.04), *P*=5.39×10^-10^ | 0.04 | 0.98 | 0.23 | 0.87 |
| **Approach 3 (PRS_Genomewide_)** |  |  |  |  |  |
| ZJCRC case-control set |  |  |  |  |  |
| Advanced lesions vs Normal | 1.70 (1.05-2.74), *P*=0.0309 | - | - | - | - |
| ZJCRC cross-sectional screening set |  |  |  |  |  |
| NAA vs Normal | 2.44 (1.31-4.53), *P*=4.68×10^-4^ | 0.03 | 0.98 | 0.40 | 0.76 |
| Advanced lesions vs NAA | 1.07 (0.43-2.67), *P*=0.888 | 0.02 | 0.98 | 0.07 | 0.81 |
| Advanced lesions vs NAA and Normal | 1.27 (0.57-2.82), *P*=0.557 | 0.02 | 0.98 | 0.04 | 0.95 |
| Advanced lesions vs Normal | 2.43 (0.81-7.23), *P*=0.112 | 0.03 | 0.98 | 0.11 | 0.93 |
| PLCO cross-sectional screening set |  |  |  |  |  |
| NAA vs Normal | 1.46 (1.17-1.83), *P*=8.56×10^-4^ | 0.03 | 0.98 | 0.24 | 0.82 |
| Advanced lesions vs NAA | 1.06 (0.87-1.28), *P*=0.583 | 0.02 | 0.98 | 0.37 | 0.6 |
| Advanced lesions vs NAA and Normal | 1.17 (0.87-1.57), *P*=0.303 | 0.02 | 0.98 | 0.13 | 0.89 |
| Advanced lesions vs Normal | 1.38 (1.14-1.67), *P*=1.04×10^-3^ | 0.02 | 0.98 | 0.15 | 0.87 |
| *Models were adjusted for age, sex, family history, genotype platform, and principal components. Two-sided *P* values per the Wald test. The error bars represent the 95% confidence intervals (CIs). OR = odds ratio. NAA, non-advanced adenoma. PPV, positive predictive value. NPV, negative predictive value. | | | | | |

| **Table S18. Prediction accuracy of three approach of the contrasted trans-ancestry PRS (10% of the PRS distribution as classifier)** | | | | | |
| --- | --- | --- | --- | --- | --- |
| Population | PRS threshold: Top 10% versus the other 90% | | | | |
|  | OR (95% CI), *P* | Sensitivity | Specificity | PPV | NPV |
| **Approach 1 (PRS_148_)** |  |  |  |  |  |
| ZJCRC case-control set |  |  |  |  |  |
| Advanced lesions vs Normal | 2.36 (1.87-2.98), *P*=4.17×10^-13^ | - | - | - | - |
| ZJCRC cross-sectional screening set |  |  |  |  |  |
| NAA vs Normal | 1.29 (0.94-1.77), *P*=0.11 | 0.11 | 0.90 | 0.28 | 0.76 |
| Advanced lesions vs NAA | 1.37 (0.74-2.55), *P*=0.318 | 0.12 | 0.90 | 0.22 | 0.82 |
| Advanced lesions vs NAA and Normal | 1.70 (1.01-2.89), *P*=0.0477 | 0.15 | 0.90 | 0.08 | 0.95 |
| Advanced lesions vs Normal | 1.85 (1.09-3.13), *P*=0.023 | 0.15 | 0.90 | 0.11 | 0.94 |
| PLCO cross-sectional screening set |  |  |  |  |  |
| NAA vs Normal | 1.08 (0.97-1.21), *P*=0.175 | 0.11 | 0.90 | 0.19 | 0.82 |
| Advanced lesions vs NAA | 1.04 (0.88-1.22), *P*=0.68 | 0.10 | 0.90 | 0.41 | 0.6 |
| Advanced lesions vs NAA and Normal | 1.08 (0.95-1.23), *P*=0.235 | 0.11 | 0.90 | 0.12 | 0.89 |
| Advanced lesions vs Normal | 1.11 (0.97-1.27), *P*=0.115 | 0.11 | 0.90 | 0.14 | 0.87 |
| **Approach 2 (PRS_183_)** |  |  |  |  |  |
| ZJCRC case-control set |  |  |  |  |  |
| Advanced lesions vs Normal | 2.39 (1.90-3.02), *P*=1.93×10^-13^ | - | - | - | - |
| ZJCRC cross-sectional screening set |  |  |  |  |  |
| NAA vs Normal | 1.53 (1.13-2.07), *P*=6.49×10^-3^ | 0.13 | 0.91 | 0.32 | 0.76 |
| Advanced lesions vs NAA | 1.28 (0.69-2.37), *P*=0.436 | 0.12 | 0.9 | 0.22 | 0.82 |
| Advanced lesions vs NAA and Normal | 1.83 (1.10-3.04), *P*=0.0197 | 0.16 | 0.9 | 0.09 | 0.95 |
| Advanced lesions vs Normal | 2.14 (1.30-3.55), *P*=2.98×10^-3^ | 0.18 | 0.91 | 0.12 | 0.94 |
| PLCO cross-sectional screening set |  |  |  |  |  |
| NAA vs Normal | 1.38 (1.24-1.54), *P*=3.5×10^-9^ | 0.13 | 0.91 | 0.23 | 0.82 |
| Advanced lesions vs NAA | 1.24 (1.05-1.46), *P*=9.96×10^-3^ | 0.11 | 0.91 | 0.45 | 0.60 |
| Advanced lesions vs NAA and Normal | 1.64 (1.46-1.85), *P*=1.64×10^-16^ | 0.14 | 0.91 | 0.16 | 0.90 |
| Advanced lesions vs Normal | 1.79 (1.59-2.01), *P*=5.91×10^-22^ | 0.15 | 0.91 | 0.20 | 0.88 |
| **Approach 3 (PRS_Genomewide_)** |  |  |  |  |  |
| ZJCRC case-control set |  |  |  |  |  |
| Advanced lesions vs Normal | 1.77 (1.41-2.21), *P*=6.29×10^-7^ | - | - | - | - |
| ZJCRC cross-sectional screening set |  |  |  |  |  |
| NAA vs Normal | 1.58 (1.17-2.15), *P*=3.18×10^-3^ | 0.13 | 0.91 | 0.32 | 0.76 |
| Advanced lesions vs NAA | 0.79 (0.39-1.60), *P*=0.514 | 0.08 | 0.89 | 0.15 | 0.81 |
| Advanced lesions vs NAA and Normal | 1.32 (0.75-2.32), *P*=0.338 | 0.12 | 0.90 | 0.06 | 0.95 |
| Advanced lesions vs Normal | 1.48 (0.84-2.60), *P*=0.171 | 0.13 | 0.90 | 0.09 | 0.93 |
| PLCO cross-sectional screening set |  |  |  |  |  |
| NAA vs Normal | 1.32 (1.19-1.47), *P*=4.09×10^-7^ | 0.12 | 0.90 | 0.22 | 0.82 |
| Advanced lesions vs NAA | 1.00 (0.85-1.18), *P*=0.966 | 0.10 | 0.90 | 0.40 | 0.60 |
| Advanced lesions vs NAA and Normal | 1.23 (1.09-1.40), *P*=1.16×10^-3^ | 0.12 | 0.90 | 0.13 | 0.89 |
| Advanced lesions vs Normal | 1.32 (1.17-1.50), *P*=1.31×10^-5^ | 0.12 | 0.90 | 0.16 | 0.87 |
| *Models were adjusted for age, sex, family history, genotype platform, and principal components. Two-sided *P* values per the Wald test. The error bars represent the 95% confidence intervals (CIs). OR = odds ratio. NAA, non-advanced adenoma. PPV, positive predictive value. NPV, negative predictive value. | | | | | |

| **Table S19. Odds ratio and covariate-adjusted AUC of ERS and PRS** | | | | | | |
| --- | --- | --- | --- | --- | --- | --- |
| Population | Best-fit PRS（PRS_183_)^a^ | | ERS^b^ | | ERS and PRS_183_ ^c^ | |
|  | AUC (crude) | OR/HR per s.d. (95% CI), *P* | AUC (crude) | OR/HR per s.d. (95% CI), *P* | AUC (crude) | OR per s.d. (95% CI), *P* |
| **ZJCRC case-control set** |  |  |  |  |  |  |
| Advanced neoplasm vs Normal | 0.607 (0.609) | 1.48 (1.38-1.58), *P*=3.20×10-^30^ | 0.581 (0.562) | 1.35 (1.26-1.44), *P*=1.22×10^-18^ | 0.629 (0.633) | 1.62 (1.51-1.74), *P*=6.43×10^-43^ |
| **ZJCRC cross-sectional screening set** |  |  |  |  |  |  |
| NAA vs Normal | 0.556 (0.558) | 1.25 (1.14-1.38), *P*=5.87×10^-6^ | 0.553 (0.592) | 1.30 (1.15-1.46), *P*=2.29×10^-5^ | 0.579 (0.575) | 1.84 (1.39-2.43), *P*=1.80×10^-5^ |
| Advanced neoplasm vs NAA | 0.567 (0.555) | 1.24 (1.02-1.52), *P*=0.0305 | 0.473 (0.503) | 0.88 (0.69-1.12), *P*=0.313 | 0.562 (0.572) | 0.76 (0.46-1.26), *P*=0.290 |
| Advanced neoplasm vs NAA and Normal | 0.586 (0.607) | 1.49 (1.24-1.80), *P*=2.14×10^-5^ | 0.500 (0.567) | 1.06 (0.86-1.3), *P*=0.575 | 0.585 (0.604) | 1.14 (0.72-1.82), *P*=0.577 |
| Advanced neoplasm vs Normal | 0.591 (0.620) | 1.61 (1.33-1.94), *P*= 8.79×10^-7^ | 0.510 (0.586) | 1.14 (0.93-1.39), *P*=0.213 | 0.589 (0.615) | 1.30 (0.84-2.17), *P*=0.215 |
| **PLCO cross-sectional screening set** |  |  |  |  |  |  |
| NAA vs Normal | 0.546 (0.550) | 1.19 (1.15-1.23), *P*=9.20×10^-23^ | 0.543 (0.547) | 1.52 (1.39-1.66), *P*=4.01×10^-21^ | 0.565 (0.568) | 1.52 (1.39-1.66), *P*= 6.40×10^-21^ |
| Advanced neoplasm vs NAA | 0.531 (0.529) | 1.13 (1.07-1.20), *P*=1.57×10^-5^ | 0.492 (0.493) | 0.94 (0.83-1.07), *P*=0.358 | 0.539 (0.527) | 0.87 (0.75-1.00), *P*=5.59×10^-2^ |
| Advanced neoplasm vs NAA and Normal | 0.570 (0.569) | 1.30 (1.24-1.35), *P*=9.66×10^-31^ | 0.529 (0.529) | 1.32 (1.19-1.46), *P*=8.59×10^-8^ | 0.575 (0.575) | 1.20 (1.12-1.39), *P*=8.07×10^-5^ |
| Advanced neoplasm vs Normal | 0.582 (0.577) | 1.34 (1.28-1.40), *P*=4.69×10^-37^ | 0.535 (0.538) | 1.42 (1.28-1.58), *P*=1.54×10^-11^ | 0.579 (0.538) | 1.34 (1.20-1.50), *P*=2.45×10^-7^ |
| **PLCO incident adenoma cohort** |  |  |  |  |  |  |
| NAA vs Normal | 0.546 (0.545) | 1.17 (1.08-1.26), *P*=4.25×10^-5^ | 0.538 (0.542) | 1.40 (1.16-1.69), *P*=4.11×10^-4^ | 0.560 (0.566) | 2.69 (1.88-3.85), *P*=6.41×10^-8^ |
| Advanced neoplasm vs Normal | 0.604 (0.591) | 1.37 (1.23-1.51), *P*=2.75×10^-9^ | 0.522 (0.538) | 1.37 (1.06-1.76), *P*=0.0166 | 0.602 (0.596) | 2.62 (1.94-3.54), *P*=3.03×10^-10^ |
| **UK Biobank cohort** |  |  |  |  |  |  |
| Advanced neoplasm vs Normal | 0.627 (0.622) | 1.57 (1.52-1.63), *P*=9.86×10^-143^ | 0.552 (0.519) | 1.35 (1.21-1.51), *P*=8.10×10^-8^ | 0.630 (0.632) | 2.67 (2.48-2.87), *P*=3.94×10^-150^ |
| Advanced neoplasm, including CRC cases and advanced adenoma. NAA, non-advanced adenoma. OR, odd ratio. HR, hazard ratio. AUC, area under curve.  ^a^ The model includes age, sex, family history, principal components, genotype platform, and continuous z-transformed PRS.  ^b^ The model includes age, sex, family history, and continuous z-transformed ERS.  ^c^ The model includes age, sex, family history, principal components, genotype platform, and continuous z-transformed PRS and ERS. | | | | | | |

| **Table S20. The additive interaction of PRS and ERS in assessment and validation set.** | | | |
| --- | --- | --- | --- |
| Population | Measure of Additive Interaction | | |
|  | RERI (95%CI) | AP (95%CI) | S (95%CI) |
| **ZJCRC case-control set** |  |  |  |
| Advanced lesions vs Normal | 0.31 [0.06, 0.58] | 0.13 [0.02, 0.23] | 1.31 [1.05, 1.65] |
| **ZJCRC cross-sectional screening set** |  |  |  |
| NAA vs Normal | 0.07 [-0.06, 0.22] | 0.05 [-0.05, 0.12] | 1.16 [0.89, 1.52] |
| Advanced lesions vs NAA | -0.02 [-0.21, 0.24] | -0.01 [-0.29, 0.12] | 0.88 [0.15, 5.18] |
| Advanced lesions vs NAA and Normal | 0.04 [-0.19, 0.33] | 0.03 [-0.18, 0.15] | 1.09 [0.68, 1.74] |
| Advanced lesions vs Normal | 0.10 [-0.15, 0.41] | 0.06 [-0.12, 0.17] | 1.15 [0.81, 1.64] |
| **PLCO cross-sectional screening set** |  |  |  |
| NAA vs Normal | 0.01 [-0.03, 0.06] | 0.01 [-0.03, 0.04] | 1.04 [0.92, 1.17] |
| Advanced lesions vs NAA | 0.00 [-0.05, 0.05] | 0.00 [-0.06, 0.04] | 0.97 [0.56, 1.68] |
| Advanced lesions vs NAA and Normal | 0.01 [-0.05, 0.07] | 0.01 [-0.04, 0.04] | 1.02 [0.89, 1.16] |
| Advanced lesions vs Normal | 0.03 [-0.03, 0.09] | 0.02 [-0.02, 0.05] | 1.05 [0.94, 1.18] |
| **PLCO incident adenoma cohort** |  |  |  |
| NAA vs Normal | 0.03 [-0.07, 0.14] | 0.02 [-0.06, 0.09] | 1.09 [0.81, 1.47] |
| Advanced neoplasm vs Normal | 0.06 [-0.09, 0.23] | 0.04 [-0.07, 0.12] | 1.11 [0.85, 1.46] |
| **UK Biobank cohort** |  |  |  |
| Advanced neoplasm vs Normal | 0.15 [0.09, 0.22] | 0.08 [0.05, 0.11] | 1.21 [1.13, 1.30] |
| * RERI, relative excess risk due to interaction; AP, attributable proportion due to interaction; S, the synergy index. | | | |

| **Table S21. Prediction accuracy of ERS and PRS-ERS strategy (20% of the ERS distribution and 2% of the PRS distribution as classifier)** | | | | | |
| --- | --- | --- | --- | --- | --- |
| Population | PRS-ERS threshold: ERS Top 20% and PRS top 2% versus other | | | | |
|  | OR/HR (95% CI), *P* | Sensitivity | Specificity | PPV | NPV |
| **ZJCRC case-control set** |  |  |  |  |  |
| Advanced neoplasm vs Normal | 6.39 (1.44-28.38), *P*=0.0148 | - | - | - | - |
| **ZJCRC cross-sectional screening set** |  |  |  |  |  |
| NAA vs Normal | 4.38 (1.03-18.73), *P*=0.046 | 0.01 | 1.00 | 0.62 | 0.75 |
| Advanced neoplasm vs NAA | 14.72 (1.49-145.66), *P*=0.0215 | 0.02 | 1.00 | 0.75 | 0.82 |
| Advanced neoplasm vs NAA and Normal | 12.77 (3.70-44.04), *P*=5.52×10^-5^ | 0.04 | 1.00 | 0.45 | 0.95 |
| Advanced neoplasm vs Normal | 22.59 (5.14-99.28), *P*=3.68×10^-5^ | 0.04 | 1.00 | 0.62 | 0.93 |
| **PLCO cross-sectional screening set** |  |  |  |  |  |
| NAA vs Normal | 1.84 (1.28-2.65), *P*=1.03×10^-3^ | 0.01 | 0.99 | 0.30 | 0.82 |
| Advanced neoplasm vs NAA | 1.41 (0.79-2.50), *P*=0.244 | 0.01 | 0.99 | 0.49 | 0.60 |
| Advanced neoplasm vs NAA and Normal | 2.19 (1.51-3.18), *P*=3.76×10^-5^ | 0.01 | 0.99 | 0.21 | 0.89 |
| Advanced neoplasm vs Normal | 2.51 (1.71-3.69), *P*=2.55×10^-6^ | 0.01 | 0.99 | 0.27 | 0.87 |
| **PLCO incident adenoma cohort** |  |  |  |  |  |
| NAA vs Normal | 1.69 (0.78-3.66), *P*=0.186 | 0.01 | 0.99 | 0.08 | 0.96 |
| Advanced neoplasm vs Normal | 2.38 (0.96-5.91), *P*=0.0621 | 0.01 | 0.99 | 0.05 | 0.98 |
| **UK Biobank cohort** |  |  |  |  |  |
| Advanced neoplasm vs Normal | 2.12 (1.37-3.27), *P*=7.05×10^-4^ | 0.06 | 0.97 | 0.05 | 0.98 |
| *Models were adjusted for age, sex, family history, genotype platform, and principal components. Two-sided *P* values per the Wald test. The error bars represent the 95% confidence intervals (CIs). Advanced neoplasm, including CRC cases and advanced adenoma. NAA, non-advanced adenoma. OR, odds ratio. HR, hazard ratio. PPV, positive predictive value. NPV, negative predictive value. | | | | | |

| **Table S22. Prediction accuracy of PRS-ERS strategy (20% of the ERS distribution and 10% of the PRS distribution as classifier)** | | | | | |
| --- | --- | --- | --- | --- | --- |
| Population | PRS-ERS threshold: ERS Top 20% and PRS top 10% versus other | | | | |
|  | OR/HR (95% CI), *P* | Sensitivity | Specificity | PPV | NPV |
| **ZJCRC case-control set** |  |  |  |  |  |
| Advanced neoplasm vs Normal | 2.65 (1.60-4.36), *P*=1.37×10^-4^ | - | - | - | - |
| **ZJCRC cross-sectional screening set** |  |  |  |  |  |
| NAA vs Normal | 2.18 (1.26-3.78), *P*=5.26×10^-3^ | 0.05 | 0.98 | 0.46 | 0.76 |
| Advanced neoplasm vs NAA | 1.69 (0.51-5.56), *P*=0.391 | 0.03 | 0.98 | 0.29 | 0.82 |
| Advanced neoplasm vs NAA and Normal | 2.59 (1.25-5.35), *P*=0.0102 | 0.08 | 0.98 | 0.16 | 0.95 |
| Advanced neoplasm vs Normal | 2.73 (1.28-5.81), *P*=9.21×10^-3^ | 0.08 | 0.98 | 0.22 | 0.94 |
| **PLCO cross-sectional screening set** |  |  |  |  |  |
| NAA vs Normal | 1.72 (1.46-2.03), *P*=1.03×10^-10^ | 0.05 | 0.97 | 0.28 | 0.82 |
| Advanced neoplasm vs NAA | 1.13 (0.88-1.45), *P*=0.341 | 0.04 | 0.96 | 0.43 | 0.6 |
| Advanced neoplasm vs NAA and Normal | 1.67 (1.38-2.01), *P*=7.85×10^-8^ | 0.05 | 0.97 | 0.17 | 0.89 |
| Advanced neoplasm vs Normal | 1.89 (1.56-2.28), *P*=4.62×10^-11^ | 0.06 | 0.97 | 0.22 | 0.87 |
| **PLCO incident adenoma cohort** |  |  |  |  |  |
| NAA vs Normal | 1.59 (1.11-2.28), *P*=0.0107 | 0.05 | 0.97 | 0.07 | 0.96 |
| Advanced neoplasm vs Normal | 2.12 (1.37-3.27), *P*=7.05×10^-4^ | 0.06 | 0.97 | 0.05 | 0.98 |
| **UK Biobank cohort** |  |  |  |  |  |
| Advanced neoplasm vs Normal | 3.07 (1.93-4.88), *P*=2.09×10^-6^ | 0.01 | 1.00 | 0.03 | 0.99 |
| *Models were adjusted for age, sex, family history, genotype platform, and principal components. Two-sided *P* values per the Wald test. The error bars represent the 95% confidence intervals (CIs). Advanced neoplasm, including CRC cases and advanced adenoma. NAA, non-advanced adenoma. OR, odds ratio. HR, hazard ratio. PPV, positive predictive value. NPV, negative predictive value. | | | | | |
