# Supplementary material for: Developing an optimal stratification model for colorectal cancer screening and reducing racial disparities in multi-center population-based studies
Source: Genome Med. 2024 Jun 13;16:81. doi: 10.1186/s13073-024-01355-y (PMC11170922; doi:10.1186/s13073-024-01355-y)
Supplement: Supplementary file 2 — Additional file 2: Supplementary Figures. Figure S1. Quality control of the genotype data of ZJCRC case-control set and ZJCRC cross-sectional screening set. Figure S2. Principal component analysis (PCA) of individuals in the assessment set and validation set and 1000 Genomes Project. Figure S3. The risk of colorectal cancer screening according to PRS&ERS categories across three assessment and validation set. Figure S4. Incident risk of colorectal neoplasm according to PRS and ERS in PLCO and UK Biobank cohort. Figure S5. Evaluation of absolute risk predictions of advanced adenoma according to PRS and ERS in UK Biobank cohort. [file 13073_2024_1355_MOESM2_ESM.pdf]

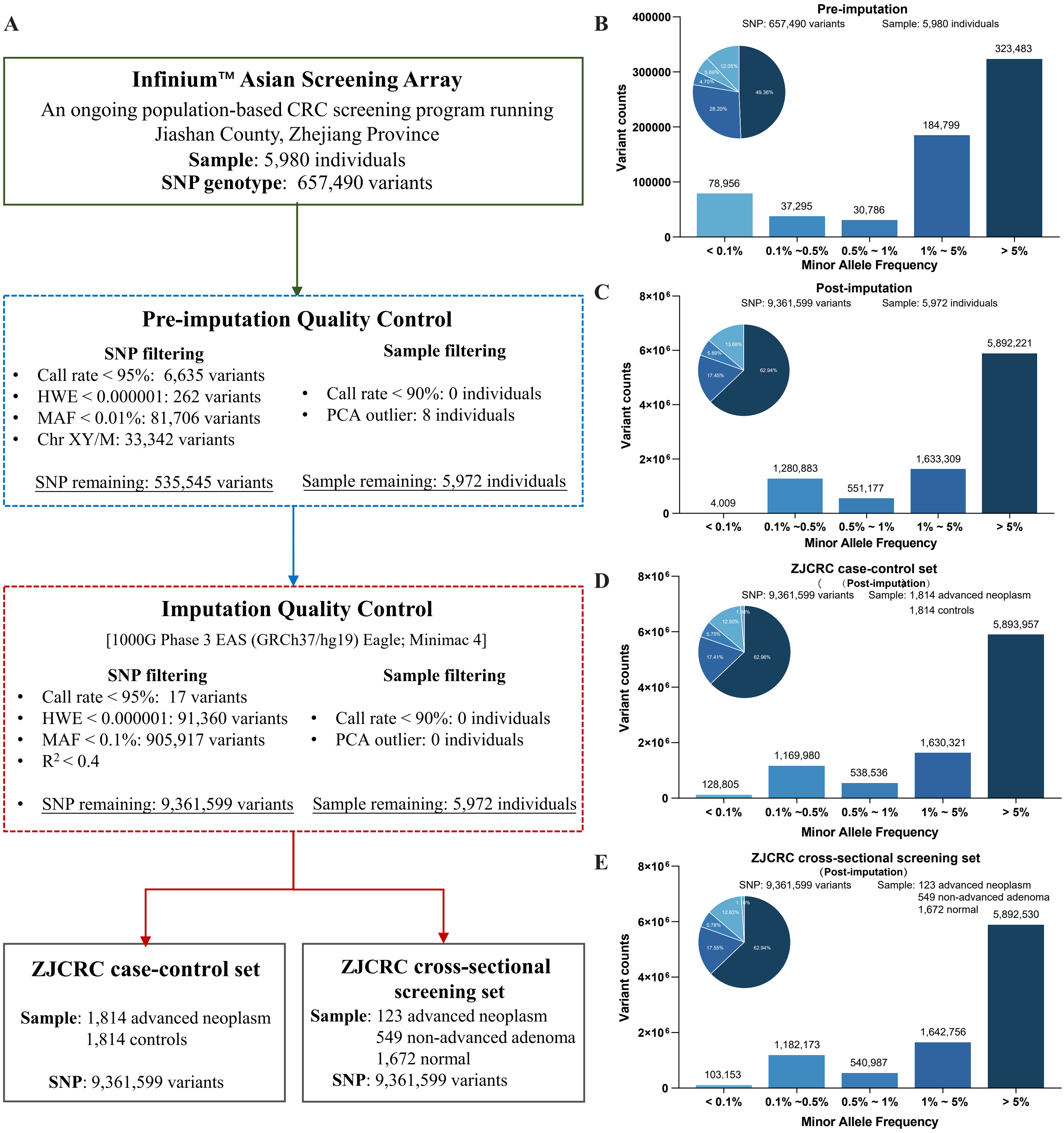

Figure S1. Quality control of the genotype data of ZJCRC case-control set and ZJCRC cross-sectional screening set. (A) The flowchart of quality control; (B-C) Variant counts at various MAF thresholds before (A) and after (B) imputation, with the proportion of variants at various MAF thresholds (%) shown in the upper left. (D-E) Variant counts at various MAF thresholds in (D) ZJCRC case-control set and (E) ZJCRC cross-sectional screening set after imputation, with the proportion of variants at various MAF thresholds (%) shown in the upper right.

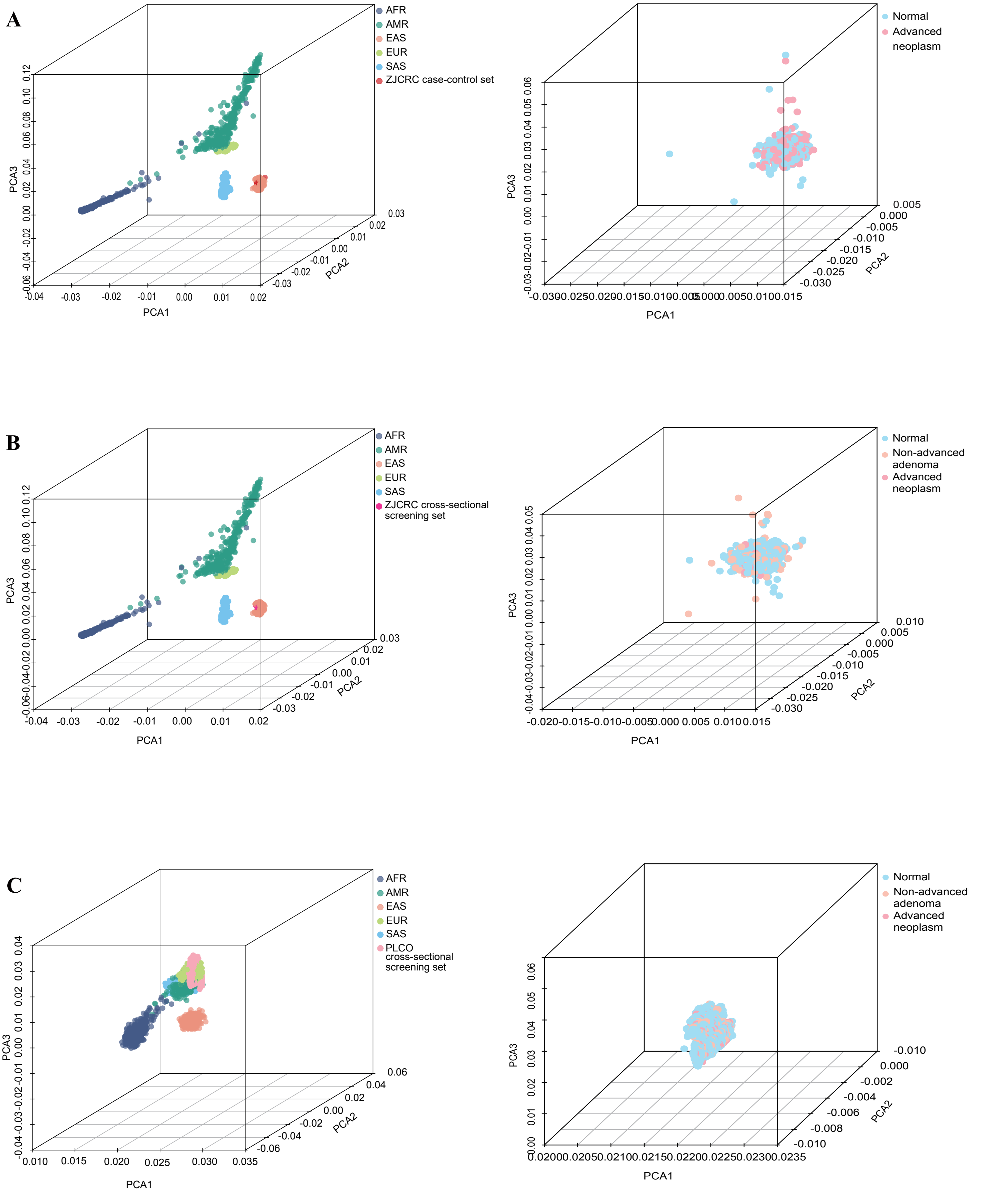

Figure S2. Principal component analysis (PCA) of individuals in the assessment set and validation set and 1000 Genomes Project. (A) ZJCRC case-control set and 1000 Genomes Project population. (B) ZJCRC cross-sectional screening set. (C) PLCO cross-sectional screening set. AFR, African; AMR,Ad Mixed American; EAS, East Asian; EUR, European; SAS, South Asian.

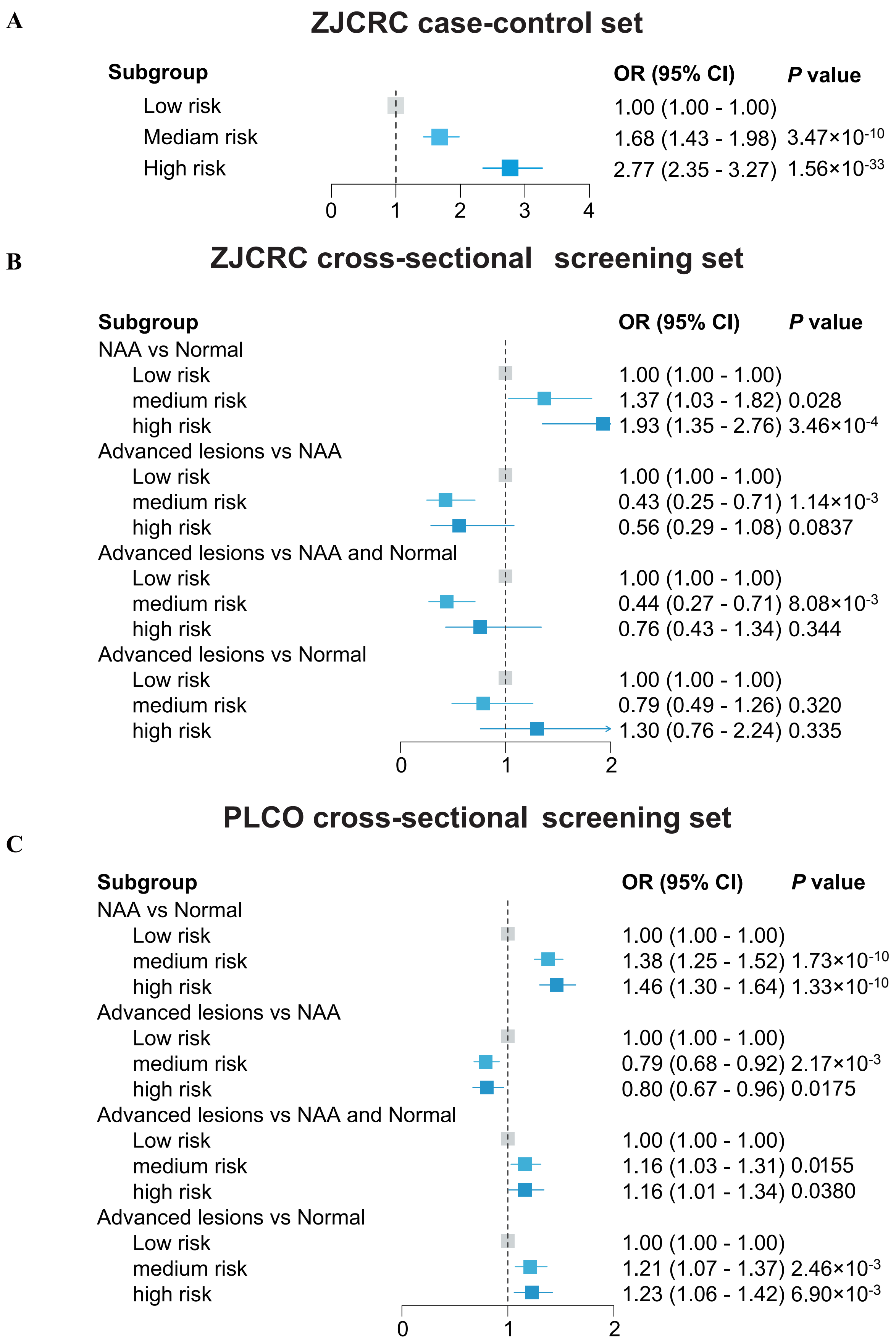

Figure S3. The risk of colorectal cancer screening according to PRS&ERS categories across three assessment and validation set. ORs for colorectal neoplasms in low, intermediate, and high risk categories according to PRS & ERS through different groups and comparisons in the ZJCRC case-control set (A), ZJCRC cross-sectional screening set (B) and PLCO cross-sectional screening set (C).

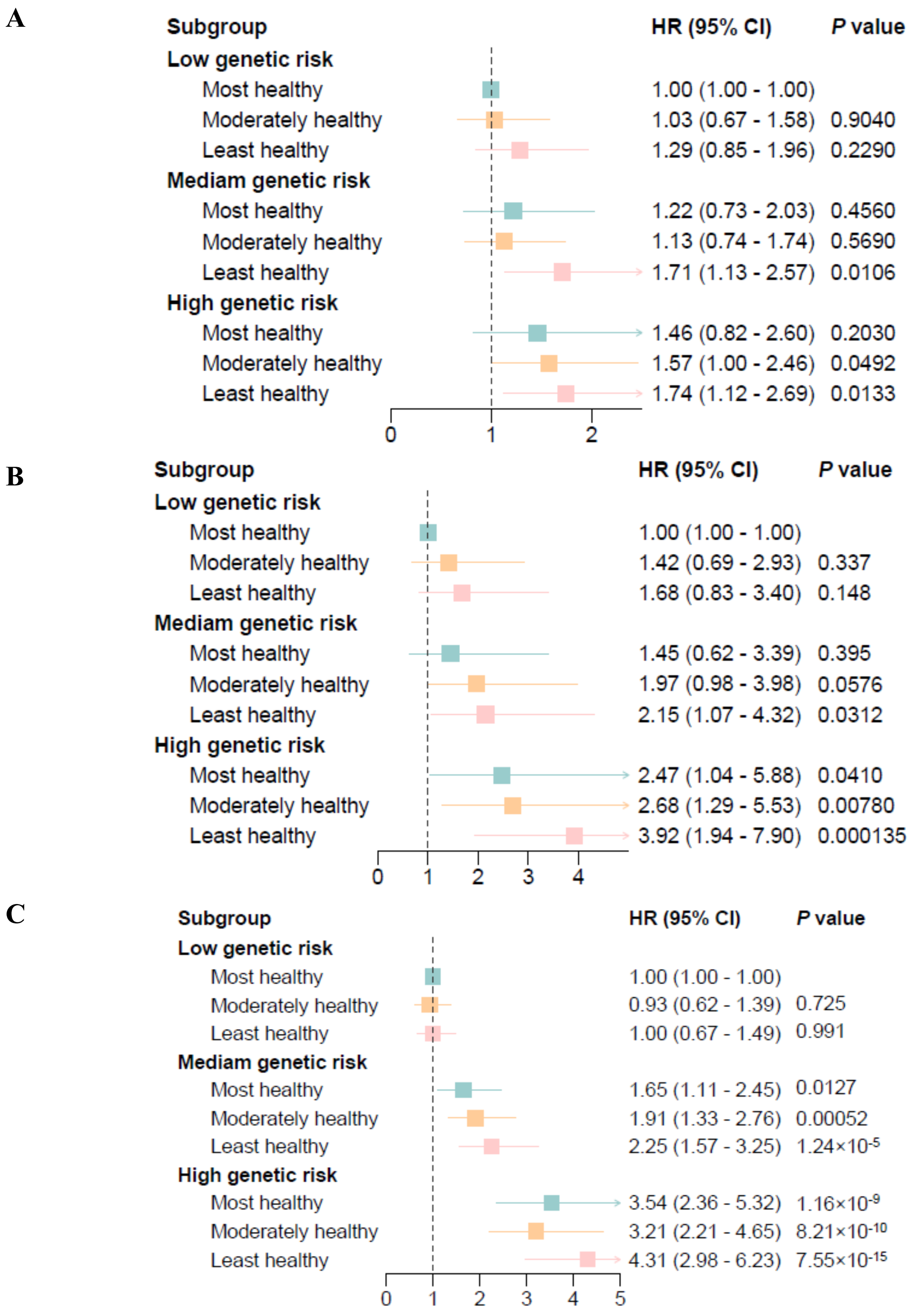

Figure S4. Incident risk of colorectal neoplasm according to PRS and ERS in PLCO and UK Biobank cohort. (A) HRs for non-advanced adenoma in low, intermediate, and high risk categories according to PRS&ERS in the PLCO incident adenoma cohort. (B) HRs for advanced neoplasm in low, intermediate, and high risk categories according to PRS&ERS through different groups and comparisons in the PLCO incident adenoma cohort. (C) HRs for advanced neoplasm in low, intermediate, and high risk categories according to PRS&ERS through different groups and comparisons in UK Biobank cohort.

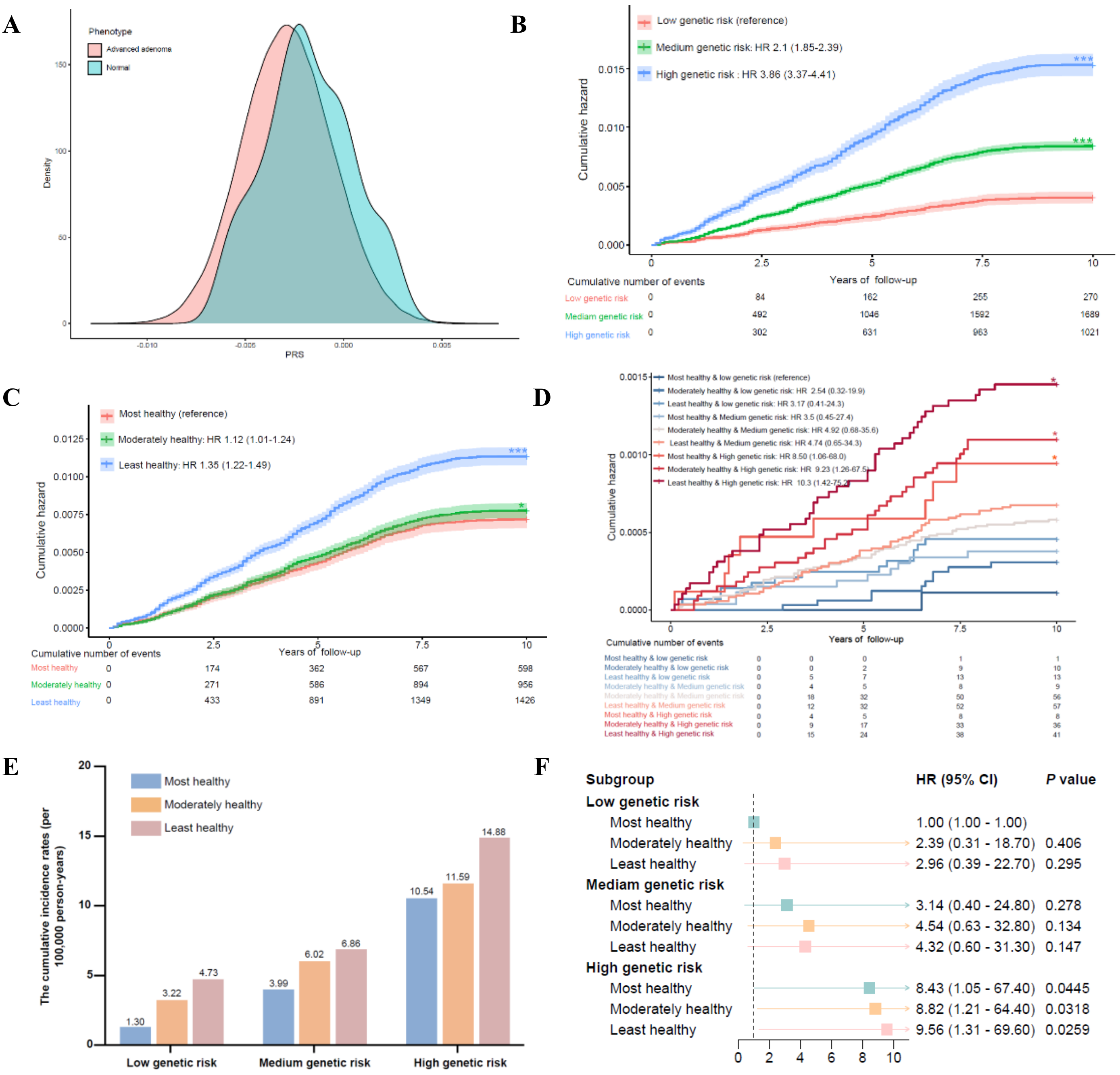

Figure S5. Evaluation of absolute risk predictions of advanced adenoma according to PRS and ERS in UK Biobank cohort. (A) Distribution of PRS. (B) Inverted Kaplan-Meier plot of incident advanced adenoma by PRS. Participants were divided into low, intermediate, and high risk groups. (C) Inverted Kaplan-Meier plot of incident advanced adenoma by ERS. Participants were divided into low, intermediate, and high risk groups. (D) Inverted Kaplan-Meier plot of incident advanced adenoma according to genetic and environmental categories. Participants were divided into 9 risk groups. The cumulative incidence of colorectal neoplasm was calculated by using the Kaplan-Meier method. The cumulative events table under the plot showed the cumulative incident events of incident colorectal neoplasm cases at years of follow-up. (E) Per 100,000 person-year at risk separately in 9 risk groups. (F) HRs for advanced adenoma in 9 risk groups according to PRS and ERS and comparisons.
